# Comparative Transcriptome Analysis of Wheat Lines in the Field Reveals Multiple Essential Biochemical Pathways Suppressed by Obligate Pathogens

**DOI:** 10.3389/fpls.2021.720462

**Published:** 2021-09-29

**Authors:** Manuel Poretti, Alexandros G. Sotiropoulos, Johannes Graf, Esther Jung, Salim Bourras, Simon G. Krattinger, Thomas Wicker

**Affiliations:** ^1^ Department of Plant and Microbial Biology, University of Zürich, Zurich, Switzerland; ^2^ Institute of Plant Sciences, University of Bern, Bern, Switzerland; ^3^ Department of Forest Mycology and Plant Pathology, Division of Plant Pathology, Swedish University of Agricultural Sciences, Uppsala, Sweden; ^4^ King Abdullah University of Science and Technology (KAUST), Biological and Environmental Science and Engineering Division (BESE), Thuwal, Saudi Arabia

**Keywords:** RNA-Seq, field trial, wheat, obligate pathogens, compatible interaction

## Abstract

Mildew and rust are the most devastating cereal pathogens, and in wheat they can cause up to 50% yield loss every year. Wheat lines containing resistance genes are used to effectively control fungal diseases, but the molecular mechanisms underlying the interaction between wheat and its fungal pathogens are poorly understood. Here, we used RNA sequencing (RNA-Seq) to compare the transcriptomic landscape of susceptible and resistant wheat lines to identify genes and pathways that are targeted by obligate biotrophic fungal pathogens. The five lines differed in the expression of thousands of genes under infection as well as control conditions. Generally, mixed infection with powdery mildew and leaf rust resulted in downregulation of numerous genes in susceptible lines. Interestingly, transcriptomic comparison between the nearly isogenic lines Thatcher and Thatcher-*Lr34* identified 753 genes that are uniquely downregulated in the susceptible line upon infection. Kyoto encyclopedia of genes and genomes (KEGG) enrichment analysis, revealed the suppression of six major biochemical pathways, namely nuclear transport, alternative splicing, DNA damage response, ubiquitin-mediated proteolysis, phosphoinositol signaling, and photosynthesis. We conclude that powdery mildew and leaf rust evade the wheat defense system by suppression of programmed cell death (PCD) and responses to cellular damage. Considering the broad range of the induced changes, we propose that the pathogen targets “master regulators” at critical steps in the respective pathways. Identification of these wheat genes targeted by the pathogen could inspire new directions for future wheat breeding.

## Introduction

Compatible interactions between host and pathogenic microbes are defined as successful infections leading to disease. In the case of obligate biotrophs (e.g., leaf rust or powdery mildew), the pathogen acquires nutrients from the host cells and therefore requires living tissues for survival. To successfully colonize a host, obligate biotrophic pathogens have evolved small secreted peptides (effectors) that are delivered into the host cells to suppress basal defense responses (Dodds et al., 2009). As defense, plants have a range of receptor proteins, for example, intracellular nucleotide-binding leucine-rich repeat proteins that can recognize effectors and induce a secondary resistance response, called effector-triggered immunity (ETI; Jones and Dangl, 2006). ETI is an intense defense response that activates several biochemical pathways, such as the production of reactive oxygen species (ROS), expression of pathogenesis-related (*PR*) genes, and also the accumulation of signaling hormones salicylic acid (SA) and jasmonic acid (JA; reviewed by de Araújo et al., 2019). ETI can culminate into a programmed cell death (PCD), also known as hypersensitive response (HR), of the infected tissues which prevents the spread of the pathogen (Dangl and Jones, 2001). Alternatively, increased accumulation of SA (Devadas and Raina, 2002) has been associated with systemic acquired resistance (SAR; Spoel and Dong, 2012). SAR is a broad-spectrum pathogen resistance response, which involves the activation of nonexpressor of pathogenesis-related genes 1 (*NPR1*) and downstream expression of several *PR* genes encoding antimicrobial proteins (Wang et al., 2006; Spoel and Dong, 2012).

Similar to SAR, broad-spectrum resistance genes are known to confer partial durable immunity against multiple fungal diseases (Ellis et al., 2014). In wheat, three broad-spectrum resistance genes (*Lr34*, Krattinger et al., 2009; *Lr46*, Singh et al., 1998; *Lr67*, Hiebert et al., 2010) were identified and their expression was shown to reduce the growth rate of fungal pathogens (i.e., partial resistance). The resistant *Lr34* allele (*Lr34res*), for example, evolved from a susceptible allele (*Lr34sus*) upon wheat domestication (Krattinger et al., 2009, 2013) and it can be transferred into other major cereals (e.g., rice or maize) to increase resistance (Chauhan et al., 2015; Krattinger et al., 2019). In recent years, the development of next-generation RNA sequencing (RNA-Seq) provided the means to broadly study the molecular and physiological changes underlying plant-pathogen interactions (Westermann et al., 2017), through comparisons of transcriptomes of infected and uninfected plants. However, even though *Lr34* has been well-studied and widely used in wheat breeding, only a few studies have investigated gene expression of cereals containing *Lr34*, and no field RNA-Seq studies has been reported in wheat. Hulbert et al. (2007) compared the wheat Thatcher/Thatcher-*Lr34* near isogenic lines (NILs) using microarray analysis, and found that 829 and 460 genes are upregulated in the tip and in the basal half of Thatcher-*Lr34* leaves, respectively. In barley, Chauhan et al. (2015) showed that 335 and 1,824 genes were differentially expressed in seedlings and mature leaves of two *Lr34res* transgenic lines, and found overexpression of several SAR-associated genes. Finally, *via* RNA-Seq of independent transgenic rice plants expressing *Lr34res*, Krattinger et al. (2019) detected 146 genes that are consistently upregulated at seedling and adult stage.

Coram et al. (2008) showed that gene expression in the host is dependent on the type of interaction (compatible vs. incompatible) and on the stage of infection. Indeed, pathogen effectors are known to reprogram the host cell’s circuitry through manipulation of the host gene expression (Durian et al., 2016). Even though rust fungi are one of the most important cereal diseases, very few studies performed transcriptomic analyses of the interaction between wheat and rust fungi. In two parallel studies, Wang et al. (2009, 2010b), identified 97 wheat genes that were downregulated in the wheat/stripe rust compatible interactions. In particular, they showed strong suppression of photosynthesis-related genes. Additionally, using a time-point infection experiment, Dobon et al. (2016) revealed that core components of the wheat innate immune response were specifically repressed during wheat/stripe rust compatible interaction, thus further suggesting that rust fungi suppress defense-related genes to colonize susceptible hosts. In summary, the previous studies showed that a wide range of genes and metabolic pathways may be affected by leaf rust infection. However, these studies were performed in the laboratory which may not fully represent the wheat transcriptomic response in the field. Furthermore, they were usually performed on only one or two wheat lines, leaving it unclear how much of the transcriptome response was line-specific.

The aim of our study was to compare transcriptomic response of different wheat lines to obligate pathogen infection and to identify wheat genes and metabolic pathways that might be targeted by powdery mildew and leaf rust. In order to have natural conditions, wheat plants (including susceptible and resistant lines) were grown in the field and infected with a mixture of fungal pathogens. Comparative transcriptome analysis allowed us to characterize differences between wheat lines and the identification of multiple crucial pathways that were affected by powdery mildew and leaf rust in the compatible interactions.

## Materials and Methods

### Field Infection Experiment, RNA Extraction, and Sequencing

Five different wheat lines (Chinese Spring, AUS 27378, AUS 27438, Thatcher and Thatcher-*Lr34*) were grown on a field near Zurich (Switzerland) and infected with a “population” of 16 Swiss wheat leaf rust isolates, as described in Messmer et al. (2000) and Singla et al. (2017). Experimental plants were grown in seven plots, each containing five rows of plants. The two outermost rows were infection rows with highly susceptible plants, while the three central rows contained the experimental plants. The order of the experimental plants was randomized across four biological replicates (Supplementary Figure S2). Control plants were grown in three plots and three replicates in a different part of the field at a distance of ~400m. The seven experimental plots formed a row of approximately 80m in length (Supplementary Figure S1). Nearby, in two parallel rows, a large experiment with powdery mildew was performed from which our experimental plants were also infected (Supplementary Figure S1). After approximately 2.5months from the infection of the spreader rows, flag leaves from infected and uninfected adult wheat plants were collected and immediately frozen in liquid nitrogen. A total of 30 field samples (5 wheat lines X 2 treatments X 3 replicates) were collected.

RNA samples were extracted using the miRNeasy kit (Qiagen), according to the manufacturer’s instructions. Quality of the extracted RNA was assessed by gel electrophoresis and with the NanoDrop ND1000 spectrophotometer based on the 260:280 ratios. The TruSeq Stranded mRNA library Preparation Kit (Illumina) was used for generating single-end-125bp read libraries, and RNA-Sequencing was carried out on Illumina HiSeq 2500 at the Functional Genomics Center Zürich.^1^ Transcriptomic sequences are available under the NCBI BioProject PRJNA718488.

### RNA-Seq Analysis

The software Salmon (Patro et al., 2017) was used in mapping-based mode (standard parameters) for quantifying the expression of wheat (IWGSC RefSeq v1.0), leaf rust (*Pt* race 1 BBBD; Cuomo et al., 2017), and powdery mildew transcripts (*B.g. tritici* 96224; Müller et al., 2019). The estimated number of mapped reads (NumReads) was retained for further analyses. First, we used the percentage of mapped reads as a proxy for estimating the abundance of wheat, powdery mildew, and leaf rust relative to each sequencing library. The average percentage of mapped reads was calculated within the biological replicates (Supplementary Figure S3). For comparison, the same analysis was done on previously published RNA-seq data of wheat seedlings infected with powdery mildew (Praz et al., 2018; Poretti et al., 2020).

As previously described by Praz et al. (2018), differential expression analysis was performed with the R package edgeR (Robinson et al., 2009). We set a minimum expression level of >5 count per million (5 CPM) in at least three RNA-Seq samples, and only the genes with a log2FC>|1.0| and an adjusted *p* value (FDR)<0.05 were considered as differentially expressed. A heatmap was used for comparing the expression of wheat genes across different lines (under uninfected condition). For each gene, we considered the three biological replicates for calculating an average Reads Per Kilobase of transcript per Million reads mapped (RPKM) value, and the R package pheatmap was used for generating the plot. In order to improve the data visualization, average RPKM values were scaled to *Z*-scores with the argument scale=“row.” Finally, we performed a KEGG enrichment analysis to further characterize the genes that were found to be downregulated in the susceptible lines Thatcher (Th) and AUS 27438 (CH59) upon pathogen infection. The KEGG orthology based annotation system (KOBAS 3.0; Bu et al., 2021) was used to annotate the protein sequence of the downregulated genes (the *Oryza sativa japonica* RefSeq database, was used as reference), to identify enriched biochemical KEGG pathways, and to visualize the results. Only KEGG pathways with an adjusted over-represented *p* value of <0.05 were considered are significantly enriched.

## Results and Discussion

Five different wheat lines (Chinese Spring, AUS 27378, AUS 27438, Thatcher and Thatcher-*Lr34*) were grown on a field near Zurich (Switzerland). Chinese Spring (hereafter CS) is the wheat reference genome, AUS 27378 (also called CHINA 15, hereafter CH15) is a leaf rust resistant landrace from China, and AUS 27438 (also called CHINA Sh59, hereafter CH59) is a highly susceptible landrace from China. Thatcher (hereafter Th) and Thatcher-*Lr34* (hereafter Th34) are two NILs. Thatcher-*Lr34* contains the durable, broad-spectrum resistance gene *Lr34res* that was transferred from the Chinese landrace PI 58548 into Thatcher through six rounds of backcrossing (Hulbert et al., 2007). Chinese Spring and CH15 also contain the *Lr34res* gene. All five lines were grown in the field in two plots; one was artificially infected with the wheat leaf rust *Puccinia triticina*, while the other was protected from infection with fungicide. Additionally, wheat powdery mildew *Blumeria graminis* f. sp. *tritici* was used for other infection experiments in nearby field plots, resulting in strong mixed infections with rust and mildew.

**Figure 1:**
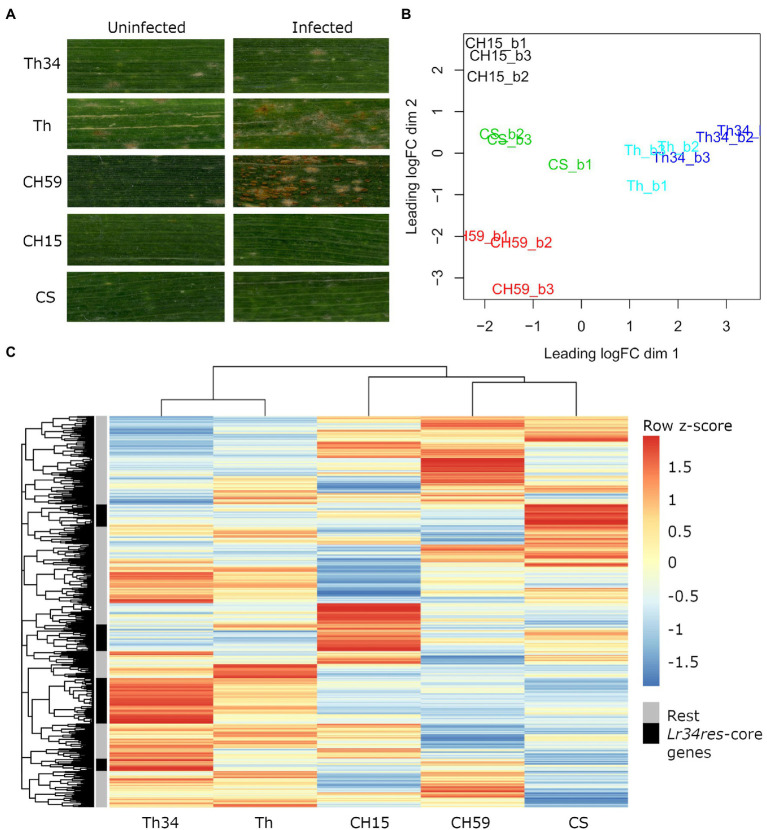
Phenotypic and transcriptomic comparison of field-grown wheat. **(A)** Flag leaves from five different wheat lines. Leaf samples were collected from uninfected and leaf rust infected wheat plants at late infection stage. **(B)** RNA sequencing (RNA-Seq) samples, each in three biological replicates, were collected from five wheat lines under uninfected conditions. Only genes with an expression value of >5cpm in at least three samples were selected. Clustering was performed with the function plot multi-dimensional scaling (MDS) from the R package edgeR. Red: CH59; green: CS; black: CH15; light blue: Th; and blue: Th34. **(C)** For each gene, we calculated the average Reads Per Kilobase of transcript per Million reads mapped (RPKM) value from the three biological replicates. The R function pheatmap was used to scale the average RPKM values into *Z*-scores and to draw the heatmap. The five most representative heatmap clusters containing the wheat homologs of the *Lr34res*-responsive core genes (see text) are highlighted in black. Note that these five clusters correspond to sets of genes that are strongly upregulated in the *Lr34res*-containing lines. Wheat lines: Thatcher (Th), Thatcher-*Lr34* (Th34), Chinese Spring (CS), AUS 27378 (CH15), and AUS 27438 (CH59).

Infected and uninfected flag leaves from the five wheat lines (adult plant stage), were collected from the field and used for phenotypic comparison and RNA-Seq. For each sample, three biological replicates were collected. As expected, all three wheat lines containing the broad-spectrum resistance gene *Lr34res* showed leaf tip necrosis, a symptom that accompanies the presence of *Lr34res* and that develops in flag leaves of adult wheat plants (Singh, 1992; Krattinger et al., 2009). Since *Lr34res* only confers partial resistance to powdery mildew and leaf rust, we expected to see at least some fungal growth. This was indeed observed in Thatcher-*Lr34* (examples in Figure 1A; Supplementary Figure S4). Interestingly, Chinese Spring and CH15 showed almost complete resistance, suggesting that other resistance genes might be present that complement the effect of *Lr34res* (examples in Figure 1A; Supplementary Figure S4). In contrast, the two susceptible wheat lines Thatcher and CH59 were both clearly susceptible and showed different degrees of susceptibility. Following the leaf rust infection scale proposed by McIntosh et al. (1995), Thatcher showed an intermediate leaf rust susceptible phenotype, with small to medium sized brown pustules (uredinia) that are sparse and usually surrounded by chlorosis, while the landrace CH59 showed more severe infection symptoms, with large numbers of uredinia without chlorosis (examples in Figure 1A; Supplementary Figure S4). Our test plants were artificially infected with leaf rust. However, when collecting the samples, we also observed advanced symptoms of powdery mildew infection in both susceptible wheat lines. Powdery mildew infects wheat earlier in the season than leaf rust, explaining the large amount of mildew spores found on the infected leaves (Supplementary Figure S4).

In order to estimate the abundance of powdery mildew and leaf rust that was present on the collected samples, we calculated the number of RNA-Seq reads mapping on the coding sequences (CDS) of both pathogens. The percentage of mapped reads was then averaged within biological replicates (Supplementary Figure S3). Overall, both pathogens showed a very similar number of mapped reads in all wheat lines, indicating that a comparable amount of fungal spores was present on the collected samples. In addition, our data confirm that both pathogens are much more abundant on infected plants than on uninfected ones. This is also true for wheat lines CH15, CS, and Thatcher-*Lr34*, thus reflecting the partial resistant phenotype conferred by the *Lr34res* allele. As expected, the largest difference of mapped fungal reads between infected and uninfected plants was found in the susceptible lines CH59 and Thatcher. In the uninfected CH59 plants, for example, only 0.52 and 0.00% of mapped reads belong to powdery mildew and leaf rust, respectively, whereas these numbers increase to ~3 and~5% upon infection. Furthermore, the percentage of mapped fungal reads in the susceptible lines reflects the degree of infection visible by eye (Figure 1A; Supplementary Figure S4). For example, Thatcher was more susceptible to powdery mildew than leaf rust, whereas CH59 shows the highest density of leaf rust uredinia.

Interestingly, the higher the number of mapped fungal reads found upon infection, the lower was the number of reads mapping to wheat CDS. This effect was particularly strong in the susceptible wheat lines CH59 and Thatcher, where only ~8 and~16% of the reads mapped to wheat CDS. This in contrast to the resistant lines, in which the number of mapped wheat reads ranged between ~27–37 and ~39–61% under control and infected conditions, respectively. These data indicate that severe infection strongly inhibits the expression of genes in the host, reflecting the overall weakening of the plants by the pathogens.

To verify that the reduction of mapped wheat reads is due to pathogen infection, we performed the same analysis using a dataset of RNA-Seq reads that were collected from mildew-infected wheat seedlings (2 and 7 dpi under laboratory-controlled conditions; Praz et al., 2018; Poretti et al., 2020). The results were comparable to those of our field experiment. In fact, compared to the uninfected control (~71% mapped wheat reads) there was a reduction of mapped wheat reads at 2 dpi (~62% mapped reads), and this reduction was even stronger at 7 dpi, with only ~14% of wheat reads mapping. Taken together, these data indicate that pathogen infection increasingly inhibits wheat gene expression over time, reflecting the overall weakening of the plants by the pathogens.

### Wheat Lines Show Very Distinct Patters of Gene Expression

The RNA-Seq analysis pipeline Salmon (Patro et al., 2017) was used for quantifying gene expression in wheat, while normalization of read counts and differential expression analysis were performed using the R package edgeR (Robinson et al., 2009). In Figure 1B, we used a multi-dimensional scaling (MDS) plot to visualize relationships between biological replicates and the different lines, and also to identify possible outliers. The distance between samples is correlated to differences in gene expression. Clustering of RNA-Seq libraries from uninfected wheat samples (Figure 1B) shows that biological replicates from the same wheat line group together well. The transcriptional differences between lines are larger than the ones within lines, clearly distinguishing the five lines, except for the NILs Thatcher and Thatcher-*Lr34* (Figure 1B). These results demonstrate the integrity of our RNA-Seq samples and show that no technical bias was introduced during field sampling, RNA extraction, and sequencing.

We used mean RPKM values that were generated from the same RNA-Seq libraries to plot a heatmap of the entire wheat genome (Figure 1C). This allows visualization and comparison of gene expression within and across lines. In addition, heatmaps can also be used for identifying a set of genes with similar expression patterns (i.e., co-expression). The output of this analysis reflects the MDS plot (Figure 1B), because even under control (uninfected) conditions, each wheat line shows a specific pattern of gene expression. Indeed, all five wheat lines differ in their gene expression patterns of thousands of genes, making each line unique (Figure 1C). Interestingly, even if the expression profiles of the NILs Thatcher and Thatcher-*Lr34* are very similar, there is a set of genes which are specifically highly differentially expressed in Thatcher-*Lr34* (Figure 1C). The resistance gene *Lr34res* is known to induce constitutive defense responses (Hulbert et al., 2007; Chauhan et al., 2015) and these genes might include homologs of “Lr34res-responsive core genes” identified in rice by Krattinger et al. (2019). However, these genes do not show a similarly high expression pattern in CH15 and CS, resistant wheat lines which also contains *Lr34res* (Figure 1C). This might be explained by the presence of uncharacterized resistance genes causing epistatic interactions, or by overall differences in genetic background of these wheat lines.

In order to verify if *Lr34res* is inducing the expression of defense-related genes in the resistant lines, we used sequence similarity search to identify wheat homologs of the previously described *Lr34res*-responsive core genes and later compared their expression profile in each line. In total, we identified 74 wheat homologs of the *Lr34res*-responsive core genes that are expressed under control conditions. Of these, 58 (78%) were specifically induced in lines containing the *Lr34res* gene (Thatcher-*Lr34*, CS, and CH15; Supplementary Table S1). This result is also reflected in the heatmap (Figure 1C). Here, we assigned the 74 expressed *Lr34res*-responsive wheat homologs to the heatmap clusters and selected the five most representative ones (Figure 1C). Indeed, we found that these five clusters exactly correspond to sets of highly expressed genes that are induced in *Lr34res*-containing lines. Therefore, we conclude that the resistance gene *Lr34res* might cause most of the transcriptional differences that are observed across lines and also activate important defense responses in the resistant lines.

### Transcriptomic Responses Differ Strongly Between Susceptible and Resistant Wheat Lines

**Figure 2:**
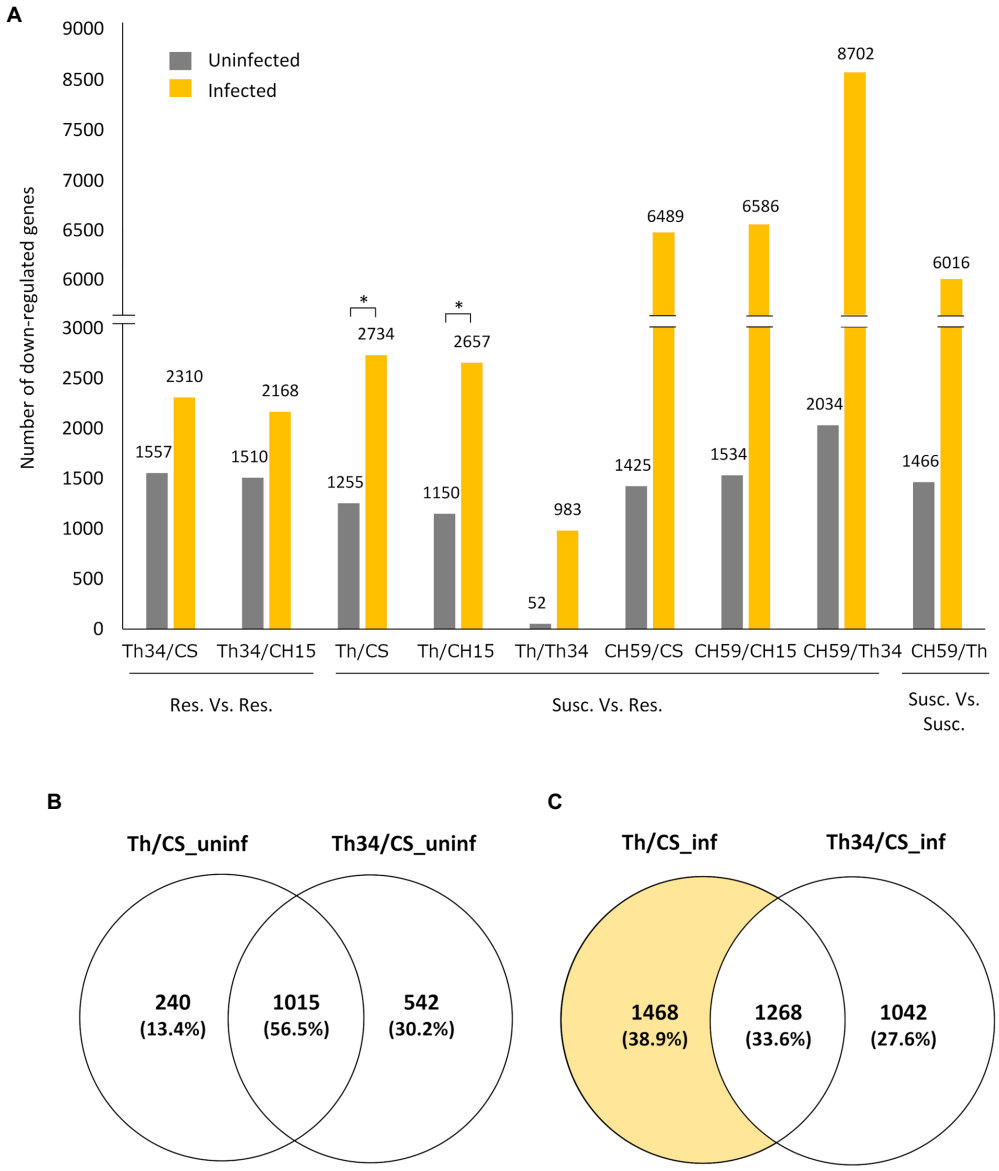
Quantitative analysis of differentially expressed genes in wheat lines. **(A)** Bar plot showing the number of genes that are downregulated (log2-fold change>|1.0|, adjusted *p* value [FDR]<0.05) between different wheat lines under control (uninfected) and infected conditions. Pairwise comparisons are indicated on the *x*-axis, and the number of genes is always referred to the first line. For example, in the comparison Thatcher/Thatcher-*Lr34*, we found that 52 and 983 genes are downregulated in Thatcher under uninfected and infected conditions, respectively. Asterisks indicate statistically significant increase of downregulated genes in Th/CS (*p*<0.05) and Th/CH15 (*p*<0.05) compared to Th34/CS and Th34/CH15, respectively, under infected conditions. **(B,C)** Venn diagrams illustrating the genes found to be downregulated in Thatcher and Thatcher-*Lr34* in comparison to Chinese Spring, under uninfected and infected conditions. Wheat lines: Thatcher (Th), Thatcher-*Lr34* (Th34), Chinese Spring (CS), AUS 27378 (CH15), and AUS 27438 (CH59).

Having three wheat lines that are at least partially resistance to obligate pathogens and two that showed different levels of susceptibility gave us the opportunity to study whether there are common transcriptomic host responses during the compatible interaction. To identify possible genes and related metabolic pathways that could be targeted by pathogen effector proteins, we performed differential expression (DE) analysis of infected wheat plants, comparing susceptible to resistant lines. Our hypothesis was that in the susceptible lines (i.e., Thatcher and CH59), the pathogen effectors would suppress the wheat immune response by inhibiting the expression of defense-related wheat genes. Therefore, we mainly focused on genes that are downregulated in the susceptible wheat lines compared to the resistant ones. Interestingly, we found that upon infection, a much higher number of genes are downregulated in susceptible lines (Figure 2A). Comparison between the Thatcher and CH15, for example, revealed that in the susceptible Thatcher line, a total of 2,657 genes are downregulated in the presence of the pathogens, whereas only 1,150 genes are suppressed under control conditions (Figure 2A). This difference is even stronger in the highly susceptible line CH59, further suggesting that obligate pathogens induce strong transcriptional reprogramming responses in the susceptible host. To support this hypothesis, we performed DE analyses between Thatcher-*Lr34* and the other two resistant lines CH15 and Chinese Spring under control and infected conditions (Figure 2A). This time, the number of downregulated genes between Thatcher-*Lr34* and the two others did not vary much under control and infected conditions. In contrast, the comparison of Thatcher with CH15 and Chinese Spring showed significantly higher number of downregulated genes upon infection (Figure 2A; Thatcher and Thatcher-*Lr34* are >99% genetically identical). We propose that this reflects the inability of the fungi to suppress genes in the resistant host.

To further validate that the downregulated genes found between susceptible and resistant lines are truly associated with the presence of the pathogens and not just the result of genomic differences between wheat lines, we compared the group of genes that are found to be downregulated in Thatcher-*Lr34* and Thatcher in comparison to Chinese Spring (Figures 2B,C). Interestingly, we found that during control (uninfected) conditions there is a much bigger overlap between these genes (1,015 genes, 56.5%; Figure 2B), whereas upon infection there is a strong increase in the number of genes (1,468 genes; Figure 2C) that are uniquely downregulated in the susceptible NIL Thatcher. Therefore, further suggesting that the transcriptional repression of host genes is caused by the pathogens.

Taken together, these results indicate that most DE genes found through comparison of susceptible vs. resistant lines, are in fact suppressed by the pathogens and therefore genuine members of candidate target pathways of pathogen effectors. Additionally, our data show that the genetic background of compared lines also has a major influence on the identification of DE genes. This is illustrated by the comparison of the NILs Thatcher and Thatcher-*Lr34* where we found only a very small number of DE genes, both in infection (983 downregulated genes) and control experiments (52 downregulated genes; Figure 2A). Near isogenic lines are genetically almost identical strains that differ only in a specific locus of the genome (Muehlbauer et al., 1988). Our NIL Thatcher-*Lr34* (R.L.6058) containing *Lr34* was derived from wheat accession PI 58548 backcrossed six times to Thatcher (Hulbert et al., 2007). Thus, the genetic background coming from PI 58548 should be less than 0.8% and purely mathematically comprise less than 900 genes. Since we were interested in the identification of genes involved in compatible interactions between wheat and obligate pathogens, we further investigated the differences between Thatcher and Thatcher-*Lr34* as is described below.

### Powdery Mildew and Leaf Rust Mixed Infection Results in the Downregulation of Hundreds of Genes in Susceptible Wheat Plants

Differential expression analysis between Thatcher and Thatcher-*Lr34* revealed that 983 and 52 wheat genes were downregulated in Thatcher upon powdery mildew and leaf rust infection and under control conditions, respectively. Of these, 40 genes were found in common between the infected and uninfected plants, whereas 12 are unique to the uninfected plants. The majority, 943 genes, were found to be downregulated only in infected Thatcher plants. These results suggest that pathogens actively suppress the transcription of defense-related genes in the susceptible wheat line. However, it is also possible that the presence of the resistance gene *Lr34* simply upregulates those 943 genes in Thatcher-Lr34 upon infection. Therefore, in order to confirm that the observed differential expression is caused by the compatible interaction between Thatcher and the pathogens, we performed a DE analysis between infected and uninfected Thatcher plants. Indeed, we found that 80% (753 out of 943) of the genes that are uniquely downregulated between the infected Thatcher and Thatcher-*Lr34*, are also downregulated in Thatcher upon infection. From this, we concluded that mixed infection with powdery mildew and leaf rust is indeed associated with the suppression of numerous host genes in the susceptible wheat line Thatcher.

**Figure 3:**
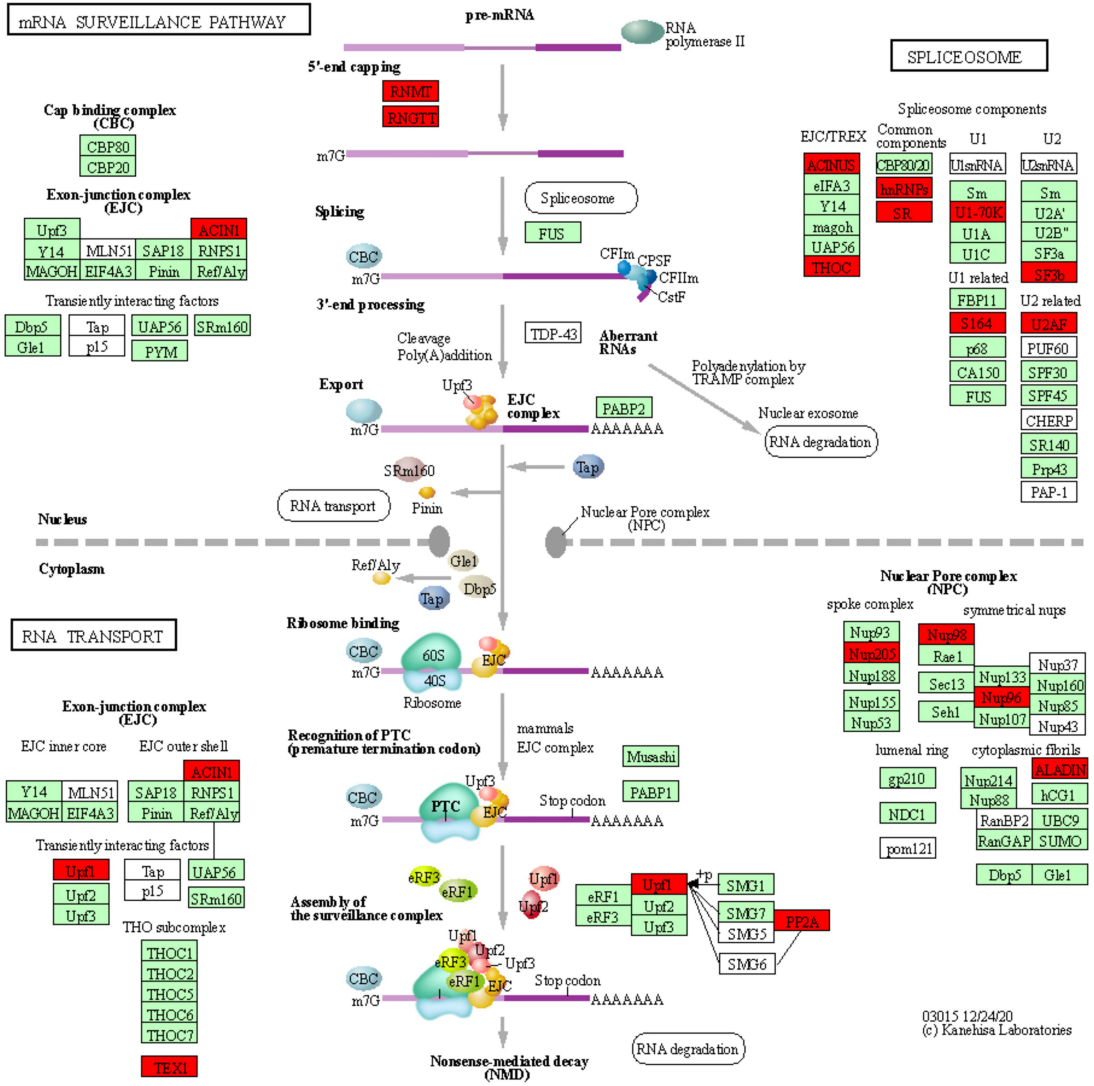
mRNA surveillance KEGG pathway. KOBAS was used to illustrate the enrichment of genes that are downregulated in Thatcher upon leaf rust infection. Osa03015 (enrichment corrected *p*-value 4.36E-02) includes two additional enriched “sub-pathways”: spliceosome (osa03040, corrected *p*-value 2.77E-02) and RNA transport (osa03013, corrected *p*-value 2.22E-03). In total, these three pathways contain 29 unique downregulated genes. Red boxes: genes that were downregulated; light green boxes: *Oryza sativa* genes that have been previously identified; white boxes: genes that belong to the KEGG pathway but have no currently identified *O. sativa* ortholog.

In order to identify defense-related wheat genes whose expression is influenced by pathogen infection, we further characterized the 753 wheat genes that were found to be specifically downregulated in Thatcher upon infection. KEGG pathway enrichment analysis revealed 69 unique downregulated genes that are significantly associated with 11 biochemical pathways (Table 1). Of particular, interest are three pathways representing mRNA surveillance (osa03015, corrected *p* value 4.36E-02), spliceosome (osa03040, corrected *p* value 2.77E-02), and RNA transport (osa03013, corrected *p* value 2.22E-03; Figure 3). These pathways are interconnected and necessary for the proper transcription of mRNA. Interestingly, we found a very similar, but more severe transcriptional response in infected CH59 wheat plants (Table 2). In total, 31 KEGG pathways were significantly enriched with 1,316 unique down-regulated genes. Of these, 451 unique genes were associated with nine out of the 11 KEGG pathways that were also enriched in Thatcher. Additionally, more closely related biochemical pathways were identified in CH59. For example, both in Thatcher and CH59, the inositol phosphate metabolism was downregulated (Tables 1 and 2), while glycerophospholipid metabolism and phosphatydilinositol signalling system were downregulated only in CH59 (Table 2). Taken together, these results not only reflect the phenotypic response that was observed in the field (Thatcher: intermediate susceptible, CH59: highly susceptible; Figure 1A; Supplementary Figure S4), but also suggest that biotrophic pathogens induce the non-specific suppression of highly conserved defense-related pathways in otherwise unrelated host lines.

**Table 1:** KEGG enrichment analysis.

ID	Downregulated genes	Total genes	Overrepresented *p* value	Adjusted *p* value	Term
osa03420	14	72	6.17E-09	5.61E-07	Nucleotide excision repair
osa03013	14	165	4.87E-05	2.22E-03	RNA transport
osa03430	7	46	1.60E-04	4.86E-03	Mismatch repair
osa04120	11	136	4.48E-04	1.02E-02	Ubiquitin mediated proteolysis
osa03030	7	60	6.92E-04	1.26E-02	DNA replication
osa03440	7	72	1.84E-03	2.77E-02	Homologous recombination
osa00195	7	75	2.28E-03	2.77E-02	Photosynthesis
osa03040	12	196	2.44E-03	2.77E-02	Spliceosome
osa00562	6	61	3.65E-03	3.69E-02	Inositol phosphate metabolism
osa03015	8	110	4.79E-03	4.36E-02	mRNA surveillance pathway
osa03022	5	47	5.76E-03	4.76E-02	Basal transcription factors

KOBAS was used for the identification of biochemical pathways that are significantly enriched (adjusted *p* value <0.05) in genes that were found to be down-regulated in the susceptible wheat line Thatcher upon leaf rust infection. The *Oryza sativa japonica* (RefSeq) protein annotation was used as reference database.

**Table 2:** KEGG enrichment analysis.

ID	Down-regulated genes	Total genes	Overrepresented *p* value	Adjusted *p* value	Term
osa01100	731	1,573	1.04E-32	5.25E-30	Metabolic pathways
osa03040^a^	128	185	4.90E-15	1.85E-12	Spliceosome
osa01110	360	848	2.54E-12	7.67E-10	Biosynthesis of secondary metabolites
osa03015^a^	80	104	1.56E-11	3.91E-09	mRNA surveillance pathway
osa01200	130	235	2.95E-10	4.45E-08	Carbon metabolism
osa00630	56	63	4.50E-10	6.16E-08	Glyoxylate and dicarboxylate metabolism
osa00970	72	113	4.68E-08	3.92E-06	Aminoacyl-tRNA biosynthesis
osa03013^a^	82	149	5.98E-07	2.37E-05	RNA transport
osa03018	59	97	2.18E-06	8.42E-05	RNA degradation
osa00195^a^	47	70	3.37E-06	1.27E-04	Photosynthesis
osa04070	34	43	6.61E-06	2.43E-04	Phosphatidylinositol signaling system
osa03430^a^	31	40	2.18E-05	7.84E-04	Mismatch repair
osa04146	48	80	2.38E-05	8.35E-04	Peroxisome
osa00710	45	73	2.60E-05	8.89E-04	Carbon fixation in photosynthetic organisms
osa04120^a^	59	109	3.02E-05	1.01E-03	Ubiquitin mediated proteolysis
osa03420^a^	41	66	5.10E-05	1.67E-03	Nucleotide excision repair
osa00510	27	34	5.43E-05	1.74E-03	N-Glycan biosynthesis
osa00010	63	123	5.80E-05	1.82E-03	Glycolysis / Gluconeogenesis
osa00051	37	58	7.77E-05	2.39E-03	Fructose and mannose metabolism
osa00906	23	27	9.23E-05	2.78E-03	Carotenoid biosynthesis
osa00640	20	22	1.45E-04	4.29E-03	Propanoate metabolism
osa00562^a^	31	47	1.86E-04	5.39E-03	Inositol phosphate metabolism
osa00030	32	51	2.85E-04	8.03E-03	Pentose phosphate pathway
osa00053	26	37	2.96E-04	8.11E-03	Ascorbate and aldarate metabolism
osa00196	15	15	5.06E-04	1.29E-02	Photosynthesis – antenna proteins
osa03022^a^	26	39	5.29E-04	1.31E-02	Basal transcription factors
osa01212	37	68	7.83E-04	1.74E-02	Fatty acid metabolism
osa00052	31	53	8.43E-04	1.84E-02	Galactose metabolism
osa00061	26	42	1.18E-03	2.43E-02	Fatty acid biosynthesis
osa00310	18	25	2.01E-03	3.98E-02	Lysine degradation
osa00564	36	71	2.32E-03	4.54E-02	Glycerophospholipid metabolism

KOBAS was used for the identification of biochemical pathways that are significantly enriched (adjusted *p* value <0.05) in genes that were found to be down-regulated in the susceptible wheat line AUS 27438 (CH59) upon leaf rust infection. The *Oryza sativa japonica* (RefSeq) protein annotation was used as reference database.

a KEGG pathways that are also found to be enriched in Thatcher (Table 1).

### RNA Processing and Nuclear Transport Are Strongly Repressed Upon Pathogen Infection

We found a total of 29 unique downregulated genes, which are mostly involved in the regulation of alternative splicing and in the formation of the nuclear pore complex (NPC; Figure 3). The NPC is a large multi-protein complex that controls gene expression through the nuclear transfer of transcription factors. As reviewed by Tamura (2020), nucleoporins Nup96 and Nup88, and importins α3 and β, regulate the expression of *PR* genes and also to trigger both PAMP- (PTI) and ETI. In addition, Cheng et al. (2009) and Roth and Wiermer (2012) demonstrated that nucleoporins Nup88, Nup160, and Seh1 are responsible for the nuclear accumulation of *NPR1* and enhanced disease susceptibility 1 (*EDS1*), which induce the expression of *PR* genes and regulate basal resistance. Furthermore, the fungal pathogen *Magnaporthe oryzae* secretes the effector AvrPiz-t which targets Nup98, thereby reducing of *PR* gene expression and increasing susceptibility of rice plants (Tang et al., 2017). Taken together, these studies indicate that nuclear transport of signaling molecules and the NPC are important for the regulation of plant immune responses and can be targeted by fungal effectors to suppress those responses. Interestingly, we also found downregulation of Nup98 and Nup96 (Figure 3), strongly suggesting that powdery mildew and leaf rust, similarly to *M. oryzae*, repress the basal innate immunity of wheat by targeting the NPC complex and consequently inhibiting the expression of *PR* genes.

In plants, alternative splicing is an important regulator of gene expression (Rigo et al., 2019) and it is known to reprogram the plant transcriptome in response to several environmental stimuli and pathogen infection (Rigo et al., 2019). Interestingly, Xu et al. (2011) showed that a member of the importin-β superfamily is involved in the nuclear transport of a serine-arginine rich protein, which is responsible for the correct splicing pattern of the two resistance genes *SNC1* and *RPS4*. These results not only highlight that alternative splicing and the nuclear pore complex are tightly coordinated in the regulation of plant immunity, but also further support the validity of our transcriptomic analysis, as we find suppression of both, the alternative splicing and NPC pathway in our data (Figure 3). In addition, several independent studies in different pathosystems showed that pathogen effectors can target components of the spliceosome complex to reprogram the plant transcriptome and therefore to subvert plant immunity (Rigo et al., 2019). Our data indicate that two components (U2AF and SF3b) of the U2 small nuclear ribonucleoprotein (snRNP) complex are downregulated in the infected Thatcher plants (Figure 3). Interestingly, the U2 snRNP is necessary for the correct alternative splicing of important regulators of plant immunity, such as the two receptor like kinases SNC4 and CERK1 (Zhang et al., 2014), thus suggesting that powdery mildew and leaf rust might target alternative splicing to repress the wheat PTI.

Type 2A protein phosphatases (PP2A) regulate stress signaling in animals and plants and also play a major role in the control of plant immune responses (Durian et al., 2016). In the enriched KEGG pathway of mRNA surveillance (osa03015, Figure 3), we found two downregulated wheat homeologs of the PP2A B′ theta subunit (TraesCS2A01G320700.2, TraesCS2D01G341400.3). Interestingly, silencing of the wheat *TaPP2Ac*, a negative regulator of ROS-scavenging enzymes CAT (Mhamdi et al., 2010) and APX2, was shown to increase the wheat resistance against the necrotrophic pathogen *Rhizoctonia cerealis* (Zhu et al., 2018). Our data suggest that biotrophs powdery mildew and leaf rust might induce the silencing of *PP2A* to reduce the accumulation of ROS in the infected tissues which could prevent hypersensitive response. Our hypothesis is substantiated by the fact that expression of PP2A B′ theta increases during senescence (Bheri and Pandey, 2019) and that both CAT and the PP2A B′ theta co-localize in the same subcellular compartments. Most importantly, Jin et al. (2016) demonstrated that the bacterial Type-III effector proteins WtsE and AvrE1 from the maize pathogen *Pantoea stewartia* subsp. *stewartii* and *Pseudomonas syringae* pv. tomato DC3000, directly target the PP2A B′ subunit.

### DNA Damage Response Is Suppressed Upon Pathogen Infection

**Figure 4:**
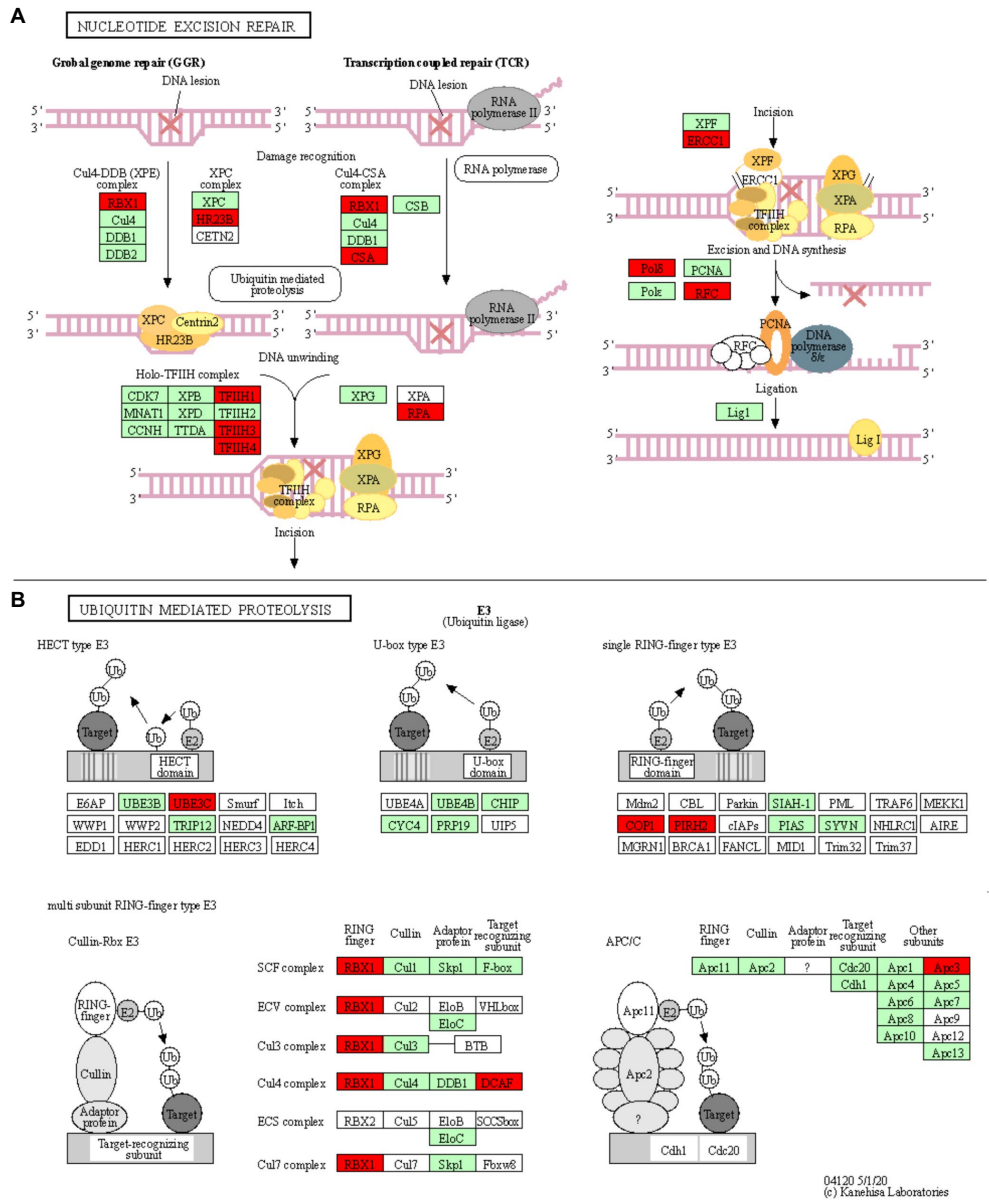
Nucleotide excision repair and ubiquitin mediated proteolysis KEGG pathways. KOBAS was used to illustrate the enrichment of genes that are downregulated in Thatcher upon leaf rust infection. **(A)** Osa03420 (enrichment corrected value of p 5.61E-07). Note that, we found four additional KEGG enriched pathways that are involved in the DNA damage repair system: osa03022, osa03430, osa03030, and osa03440 (see Table 1). In total, these five pathways contain 18 unique downregulated genes. **(B)** Osa04120 (enrichment corrected value of p 1.02E-02). Note that most of down-regulated genes are E3 ubiquitin ligases. In total, this pathway contains 11 unique downregulated genes. Red boxes: genes that were downregulated; light green boxes: *O. sativa* genes that have been previously identified; and white boxes: genes that belong to the KEGG pathway but have no currently identified *O. sativa* ortholog.

Five enriched KEGG pathways (osa03420, osa03430, osa03440, osa03030, and osa03022) are part of the plant DNA Damage Response and are associated with a total of 18 downregulated unique genes (Table 1; Figure 4A; Supplementary Figures S5–S8). Surprisingly, our results show that most of the wheat repair machineries (mismatch repair, Nucleotide Excision Repair, NER, and homologous recombination) are transcriptionally repressed in Thatcher upon leaf rust infection (Table 1). Furthermore, five out of six steps in the NER pathway (damage recognition, DNA unwinding, incision, excision, and DNA synthesis) are transcriptionally silenced (Figure 4A). The DNA damage response was proposed to contribute to the plant immune response either by limiting the plant cell death due to accumulation of DNA lesions or, by inducing PCD (Nisa et al., 2019). In addition, during pathogen infection, members of the homologous recombination pathways (RAD51 and BRCA2) can directly bind to the promoter of *PR* defense genes and induce their expression (Wang et al., 2010a) For these reasons, we suggest that powdery mildew and leaf rust, similar to plant bacterial pathogens, actively target the DNA damage response pathway of wheat to suppress the activation of PCD and the transcription of defense genes.

### Ubiquitin-Mediated Proteolysis Is Affected *via* Suppression of E3 Ubiquitin Ligases

Another interesting KEGG enriched pathway is the Ubiquitin mediated proteolysis (osa04120, corrected *p* value 1.02E-02; Table 1). Most of the downregulated genes are associated with E3 ubiquitin ligases from the HECT, RING, and cullin-RING subfamilies (Figure 4B). E3 enzymes ligate ubiquitin molecules to specific target proteins, which are finally degraded by the proteasome. Recent publications have shown that ubiquitin-mediated protein degradation plays a major role in the control of the plant immune response (Furniss et al., 2018). Especially RING proteins have been shown to positively regulate the production of ROS and to induce HR/ETI responses by regulating the protein level of NLR receptors and RLKs (Marino et al., 2012). Therefore, we suggest that powdery mildew and leaf rust might suppress the immune response of wheat by downregulating the activity of E3 ligases, thereby suppressing PCD. Indeed, *M. oryzae* and pathogenic bacteria have been shown to directly suppress or hijack the ubiquitin proteasome system, in order to degrade defense-related components of the host (Park et al., 2012).

### Obligate Pathogens Might Disrupt Early Defense Responses by Suppression of the PI-PLC Signaling Pathway

The phosphatidylinositol phospholipase C (PI-PLC)-mediated hydrolysis of inositol phospholipids (phosphoinositides, PIs) plays a major role in the initiation of defense responses (Abd-El-Haliem and Joosten, 2017). In fact, PI-PLC can be activated by the stress hormone ABA and different transmembrane immune receptors (e.g., *Cf*-4 and FLS2) to trigger hypersensitive response (Abd-El-Haliem and Joosten, 2017). Interestingly, we found a significant enrichment of downregulated genes in the inositol phosphate metabolism (KEGG pathway osa00562, corrected *p* value 3.69E-02; Table 1), including the transcriptional repression of PI-PLC (EC 3.1.4.11, Supplementary Figure S9). This indicates that during the compatible interaction between wheat, mildew, and rust, the transcriptional silencing of PI-PLC might play an important role in the repression HR-triggered immunity. Indeed, fungal pathogens have been shown to manipulate the inositol phosphate metabolic pathway to escape NB-LRR recognition and to increase the virulence function of the effectors (Kale et al., 2010). The *Phytophthora infestans* Avr3a protein, for example, binds to PI-PLC substrates PI3P, PI4P, and PI5P, in order to increase its stability and concentration in the host cell (Yaeno et al., 2011).

### Powdery Mildew and Leaf Rust Infection Might Induce Repression of Chloroplastic Photosystems

Finally, we found an overrepresentation of downregulated genes in the photosynthesis pathways (osa00195, corrected *p* value 2.77E-02; Table 1). The chloroplast was suggested to be a key component of early immune response. It integrates multiple environmental stimuli, such as PAMP-triggered Ca^2+^ and ABA signals, to induce the formation of ROS and subsequent expression of defense genes, through retrograde signaling to the nucleus (Göhre, 2015). Interestingly, de Torres Zabala et al. (2015) showed that virulent *Pseudomonas syringae* effectors inhibit the chloroplastic ROS burst in *Arabidopsis* through disruption of the photosystem II. Similarly, our data show that most part of the wheat photosynthetic machinery (photosystems I and II, cytochrome b6/f complex, and the ATP synthase; Supplementary Figure S10) is repressed upon infection, specifically in the susceptible lines. For these reasons, we suggest that the chloroplast might be an important conserved target for the manipulation of plant immune responses.

## Conclusion

Comparative transcriptome analysis of five wheat lines revealed striking differences between lines, both in infected and control plants. To our knowledge, this is the first field study on the wheat transcriptomic response during compatible interaction with the obligate pathogens powdery mildew and leaf rust. Our experimental set up allowed comparisons of resistant and susceptible lines, as well as comparisons of susceptible lines under control and infection conditions. Most striking to us was how broadly, and yet how specifically, obligate pathogens influence the transcriptional landscape of its host. On one hand, the pathogens suppress the most potent plant defense, namely PCD, by repressing pivotal elements such as ROS production. On the other hand, the pathogens target the host systems that recognize and respond to cellular damage (DNA repair and protein degradation), thereby leaving the plant cell essentially blind to the pathogen presence.

Previous studies have shown that obligate biotrophic pathogens have an arsenal of hundreds of effector proteins which they can deliver into the plant (Schwessinger et al., 2018; Müller et al., 2019). These effectors can be grouped into just a few major protein families (Müller et al., 2019). This suggests functional redundancy, and that effector protein families may actually target a relatively small number of host proteins. Considering how broad the impact of a compatible interaction is on the host transcriptome, effector targets are probably “master regulator” proteins at critical steps of the targeted pathways. Identification of these effector targets will be essential for our understanding of the molecular dynamics underlying the compatible interaction between wheat and obligate pathogens, such as mildew and rust fungi. As demonstrated by Wang et al. (2014), such knowledge could be used in wheat breeding for the targeted mutagenesis of effector targets that may be essential for the virulence of the fungus.

## Data Availability Statement

The original contributions presented in the study are publicly available. This data can be found here: NCBI repository, BioProject accession number: PRJNA718488.

## Author Contributions

MP and TW wrote the manuscript, designed the study, and coordinated the research. MP, TW, SK, and SB edited the manuscript. TW and SK designed the field experiment. EJ coordinated the field experiment. MP, TW, and SB collected field samples and generated sequencing material. MP, AS, and JG performed bioinformatic analyses. All authors contributed to the article and approved the submitted version.

## Conflict of Interest

The authors declare that the research was conducted in the absence of any commercial or financial relationships that could be construed as a potential conflict of interest.

## Publisher’s Note

All claims expressed in this article are solely those of the authors and do not necessarily represent those of their affiliated organizations, or those of the publisher, the editors and the reviewers. Any product that may be evaluated in this article, or claim that may be made by its manufacturer, is not guaranteed or endorsed by the publisher.

## Footnotes

^1^ http://www.fgcz.ch/
