# Supplementary material for: Comparative Transcriptome Analysis of Wheat Lines in the Field Reveals Multiple Essential Biochemical Pathways Suppressed by Obligate Pathogens
Source: Front Plant Sci. 2021 Sep 29;12:720462. doi: 10.3389/fpls.2021.720462 (PMC8513673; doi:10.3389/fpls.2021.720462)
Supplement: Supplementary file 1 [file Data_Sheet_1.docx]

Supplementary Material

**Supplementary table S1.** Expressed wheat *Lr34res*-responsive core genes. Wheat homologous genes were found by Blastn analysis of the rice *Lr34res*-responsive core genes (Krattinger et al. 2019) against the wheat CDS (RefSeq 1.0). Best hits were selected and checked for expression. Genes with an expression level of > 5 CPM (count per million) in at least 3 RNA-Seq samples were considered as expressed. The average RPKM values from the three biological replicates are shown in the table. The last column highlights the cultivar in which the gene is more expressed.

| **Gene_ID** | **CH15** | **CH59** | **CS** | **Th34** | **Th** | **Highly expressed** |
| --- | --- | --- | --- | --- | --- | --- |
| TraesCS1A01G073300 | 4,86 | 2,38 | 2,45 | 3,35 | 2,73 | CH15 |
| TraesCS1A01G299100 | 12,98 | 9,21 | 7,58 | 15,74 | 10,84 | Th34 |
| TraesCS1B01G091900 | 3,27 | 2,38 | 4,00 | 1,11 | 2,49 | CS |
| TraesCS1B01G248600 | 6,94 | 2,71 | 7,02 | 1,47 | 3,73 | CS |
| TraesCS1B01G308900 | 73,76 | 12,74 | 20,67 | 37,89 | 23,77 | CH15 |
| TraesCS1D01G076200 | 7,83 | 4,29 | 7,87 | 2,96 | 5,04 | CS |
| TraesCS1D01G292100 | 13,61 | 0,00 | 7,32 | 35,96 | 14,43 | Th34 |
| TraesCS2A01G054200 | 0,65 | 12,90 | 23,65 | 11,65 | 16,07 | CS |
| TraesCS2A01G152400 | 11,89 | 6,56 | 8,65 | 6,36 | 7,19 | CH15 |
| TraesCS2A01G219100 | 16,31 | 0,00 | 4,66 | 0,05 | 1,57 | CH15 |
| TraesCS2A01G277100 | 3,65 | 2,65 | 2,79 | 7,03 | 4,16 | Th34 |
| TraesCS2A01G345500 | 55,06 | 0,10 | 20,95 | 11,05 | 10,70 | CH15 |
| TraesCS2A01G350700 | 0,68 | 3,26 | 0,37 | 6,38 | 3,34 | Th34 |
| TraesCS2A01G467700 | 13,80 | 0,87 | 2,46 | 1,19 | 1,51 | CH15 |
| TraesCS2A01G509600 | 23,70 | 21,98 | 26,38 | 22,40 | 23,59 | CS |
| TraesCS2A01G556100 | 5,62 | 1,30 | 2,95 | 5,61 | 3,29 | CH15 |
| TraesCS2B01G272900 | 105,12 | 134,23 | 18,00 | 178,39 | 110,20 | Th34 |
| TraesCS2B01G294500 | 13,86 | 12,83 | 11,59 | 59,05 | 27,82 | Th34 |
| TraesCS2B01G364000 | 22,55 | 0,06 | 4,34 | 1,47 | 1,96 | CH15 |
| TraesCS2B01G369000 | 7,56 | 21,64 | 6,79 | 14,67 | 14,36 | CH59 |
| TraesCS2D01G157800 | 5,51 | 5,77 | 2,82 | 5,97 | 4,85 | Th34 |
| TraesCS2D01G276000 | 3,94 | 0,00 | 1,68 | 14,89 | 5,52 | Th34 |
| TraesCS2D01G344200 | 102,84 | 0,19 | 19,95 | 4,93 | 8,36 | CH15 |
| TraesCS3A01G122100 | 7,19 | 11,25 | 10,14 | 10,13 | 10,51 | CH59 |
| TraesCS3A01G225100 | 0,74 | 4,81 | 2,64 | 0,27 | 2,58 | CH59 |
| TraesCS3A01G517100 | 183,66 | 123,48 | 33,64 | 518,43 | 225,19 | Th34 |
| TraesCS3B01G141300 | 5,75 | 9,42 | 8,70 | 10,34 | 9,49 | Th34 |
| TraesCS3B01G254700 | 1,89 | 8,13 | 2,70 | 0,29 | 3,71 | CH59 |
| TraesCS3B01G293200 | 1,83 | 7,62 | 1,05 | 35,30 | 14,66 | Th34 |
| TraesCS3B01G396900 | 3,59 | 6,75 | 4,48 | 4,68 | 5,31 | CH59 |
| TraesCS3B01G584700 | 54,87 | 31,35 | 6,68 | 71,32 | 36,45 | Th34 |
| TraesCS3D01G358200 | 6,36 | 16,91 | 7,57 | 5,55 | 10,01 | CH59 |
| TraesCS3D01G475000 | 7,85 | 12,50 | 6,83 | 33,11 | 17,48 | Th34 |
| TraesCS3D01G524700 | 37,96 | 231,49 | 56,73 | 99,75 | 129,32 | CH59 |
| TraesCS4A01G233600 | 17,58 | 10,23 | 27,19 | 9,41 | 15,61 | CS |
| TraesCS4A01G484800 | 2,10 | 4,79 | 4,04 | 1,16 | 3,33 | CH59 |
| TraesCS4B01G082100 | 10,47 | 9,43 | 13,58 | 9,88 | 10,96 | CS |
| TraesCS4B01G205700 | 5,71 | 4,22 | 6,45 | 18,41 | 9,69 | Th34 |
| TraesCS4B01G251100 | 10,21 | 3,27 | 8,57 | 2,10 | 4,64 | CH15 |
| TraesCS4D01G080600 | 18,09 | 14,25 | 27,36 | 19,15 | 20,25 | CS |
| TraesCS4D01G136600 | 2,67 | 5,13 | 3,30 | 1,38 | 3,27 | CH59 |
| TraesCS4D01G251000 | 12,54 | 3,20 | 7,65 | 1,58 | 4,14 | CH15 |
| TraesCS4D01G299800 | 23,54 | 27,04 | 15,79 | 29,19 | 24,01 | Th34 |
| TraesCS5A01G091400 | 7,79 | 11,85 | 8,28 | 7,90 | 9,34 | CH59 |
| TraesCS5A01G143200 | 7,48 | 0,46 | 2,17 | 0,81 | 1,15 | CH15 |
| TraesCS5A01G402300 | 16,84 | 17,54 | 26,09 | 20,37 | 21,34 | CS |
| TraesCS5B01G083700 | 13,78 | 5,96 | 7,51 | 44,62 | 19,36 | Th34 |
| TraesCS5B01G098100 | 14,06 | 20,31 | 14,42 | 13,77 | 16,16 | CH59 |
| TraesCS5B01G141900 | 4,48 | 4,60 | 2,99 | 4,43 | 4,01 | CH59 |
| TraesCS5B01G142100 | 6,47 | 0,52 | 2,44 | 2,37 | 1,78 | CH15 |
| TraesCS5B01G407200 | 7,75 | 0,55 | 2,84 | 3,30 | 2,23 | CH15 |
| TraesCS5D01G090100 | 4,79 | 5,81 | 3,81 | 9,46 | 6,36 | Th34 |
| TraesCS5D01G412400 | 11,68 | 21,52 | 20,30 | 12,46 | 18,09 | CH59 |
| TraesCS5D01G415700 | 13,69 | 10,85 | 9,49 | 8,04 | 9,46 | CH15 |
| TraesCS5D01G455900 | 3,76 | 3,94 | 5,58 | 4,94 | 4,82 | CS |
| TraesCS6A01G058400 | 40,22 | 33,44 | 52,90 | 32,98 | 39,77 | CS |
| TraesCS6A01G170700 | 1,48 | 5,56 | 3,12 | 3,48 | 4,05 | CH59 |
| TraesCS6A01G240400 | 8,78 | 4,34 | 12,20 | 5,34 | 7,29 | CS |
| TraesCS6A01G344000 | 41,44 | 8,65 | 13,66 | 13,83 | 12,05 | CH15 |
| TraesCS6A01G374400 | 1932,09 | 944,69 | 2064,24 | 2142,30 | 1717,08 | Th34 |
| TraesCS6B01G031700 | 2,14 | 0,63 | 15,39 | 6,18 | 7,40 | CS |
| TraesCS6B01G074100 | 8,43 | 7,88 | 8,78 | 12,89 | 9,85 | Th34 |
| TraesCS6B01G376200 | 3,28 | 2,01 | 0,00 | 0,00 | 0,67 | CH15 |
| TraesCS6D01G057800 | 195,39 | 101,41 | 97,27 | 212,16 | 136,95 | Th34 |
| TraesCS6D01G325100 | 39,87 | 7,98 | 4,82 | 9,03 | 7,28 | CH15 |
| TraesCS6D01G358900 | 4245,79 | 2500,64 | 4420,82 | 4410,79 | 3777,42 | CS |
| TraesCS7A01G306600 | 4,16 | 4,58 | 2,78 | 8,43 | 5,26 | Th34 |
| TraesCS7A01G454600 | 20,68 | 14,59 | 17,33 | 36,95 | 22,96 | Th34 |
| TraesCS7A01G479700 | 1,47 | 9,48 | 5,64 | 2,68 | 5,93 | CH59 |
| TraesCS7B01G206900 | 3,74 | 4,48 | 3,58 | 24,24 | 10,77 | Th34 |
| TraesCS7B01G355200 | 35,91 | 15,09 | 18,80 | 47,99 | 27,29 | Th34 |
| TraesCS7B01G382000 | 0,95 | 7,24 | 0,21 | 2,41 | 3,29 | CH59 |
| TraesCS7D01G010000 | 3,19 | 3,97 | 4,31 | 15,76 | 8,01 | Th34 |
| TraesCS7D01G303500 | 8,13 | 9,08 | 7,84 | 60,66 | 25,86 | Th34 |


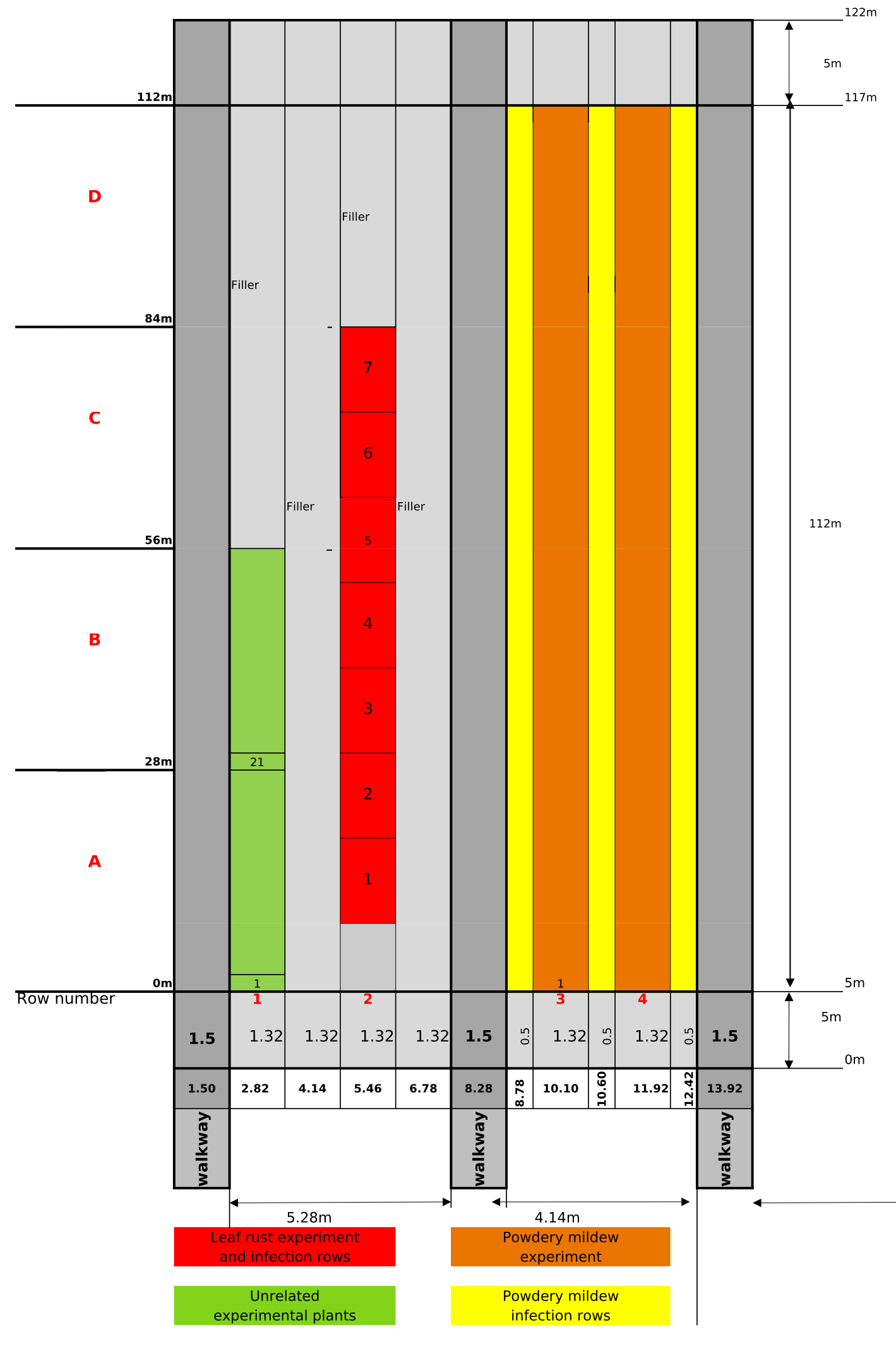


**Supplementary fig. S1. Set up of the experimental field**. The seven experimental plots (highlighted in red) formed a row of approximately 80 metres in length. Nearby, in two parallel rows, a large experiment with powdery mildew was performed from which our experimental plants were also infected.


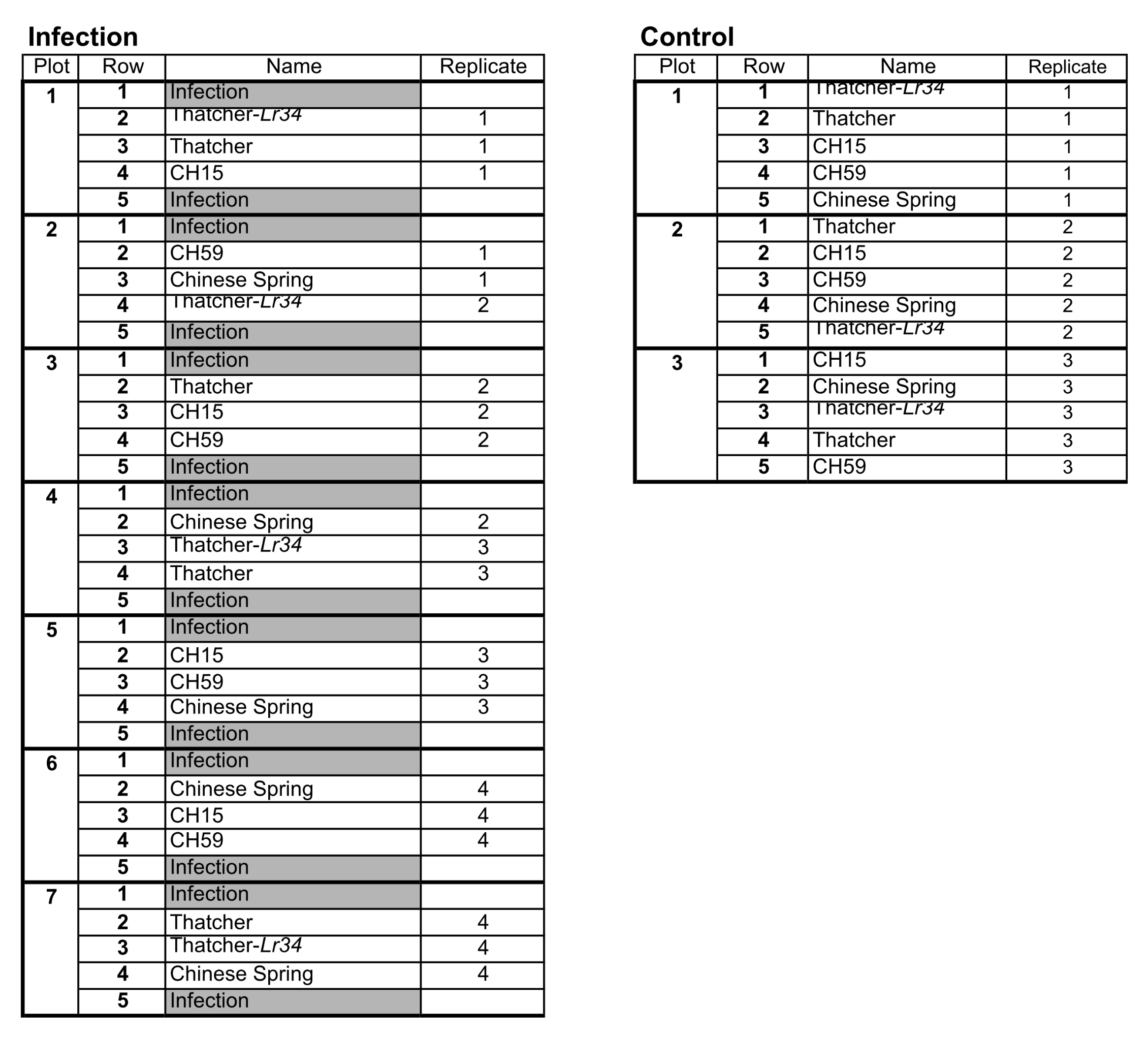


**Supplementary fig. S2.** **Field infection design.** Experimental plants were grown in seven plots, each containing five rows of plants. The two outermost rows (i.e., highly susceptible spreader rows) were artificially infected, while the three central rows contained the experimental plants. The order of the experimental plants was randomized across four biological replicates. Control plants were grown in three plots and three replicates in a different part of the field at a distance of ~400 m. Wheat lines: Thatcher (Th), Thatcher-*Lr34* (Th34)*,* Chinese Spring (CS), AUS 27378 (CH15), AUS 27438 (CH59).


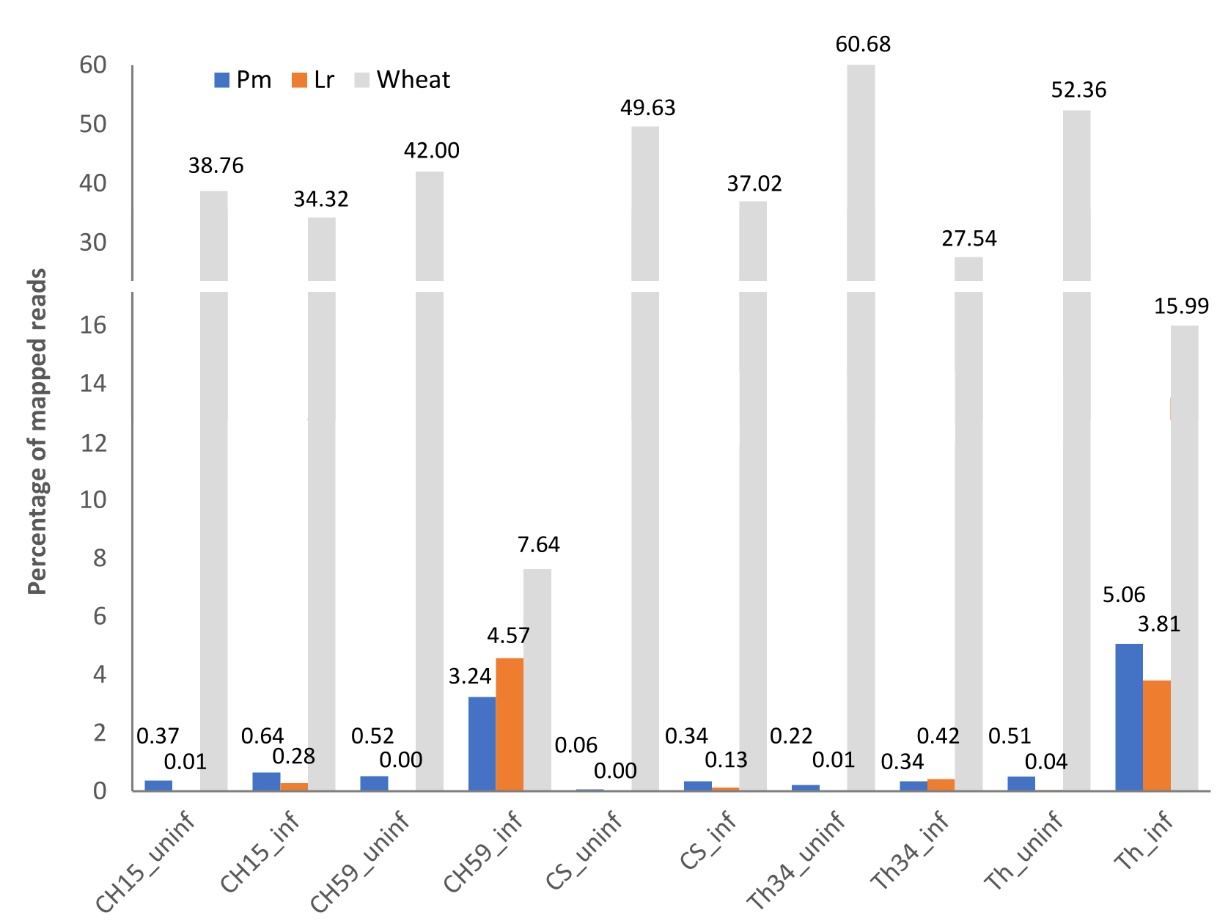


**Supplementary fig. S3.** **Estimation of pathogen abundance.** The software Salmon was used for estimating the number of reads that map to the coding sequences of wheat, powdery mildew (Pm) and leaf rust (Lr), separately. For each species, we consider the percent of mapped reads relative to each sequencing library for estimating the abundance of the fungal pathogens on the collected samples. The average percentage of mapped reads was calculated within the biological replicates and used for visualizing the data. Wheat lines: Thatcher (Th), Thatcher-*Lr34* (Th34)*,* Chinese Spring (CS), AUS 27378 (CH15), AUS 27438 (CH59).


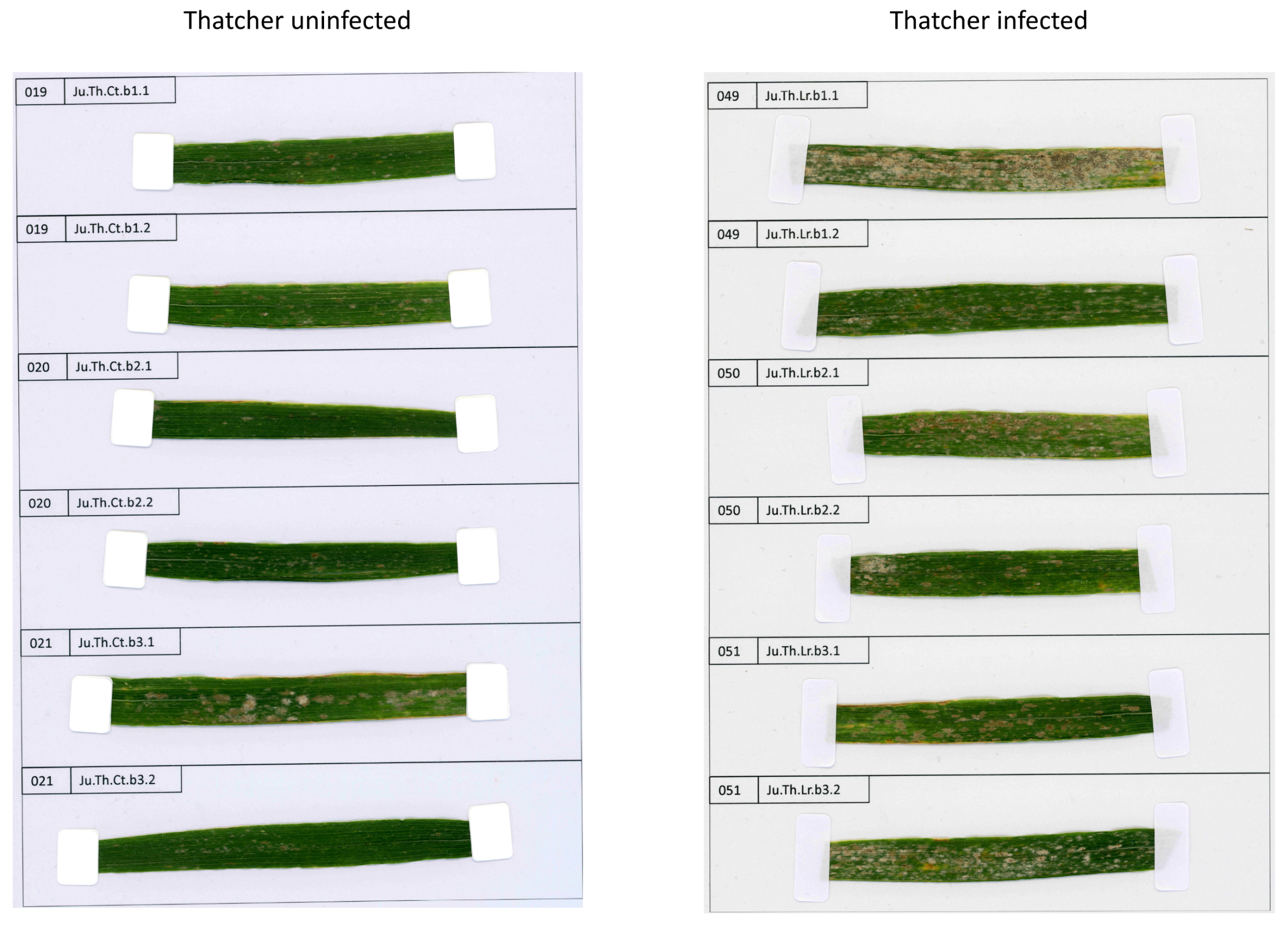


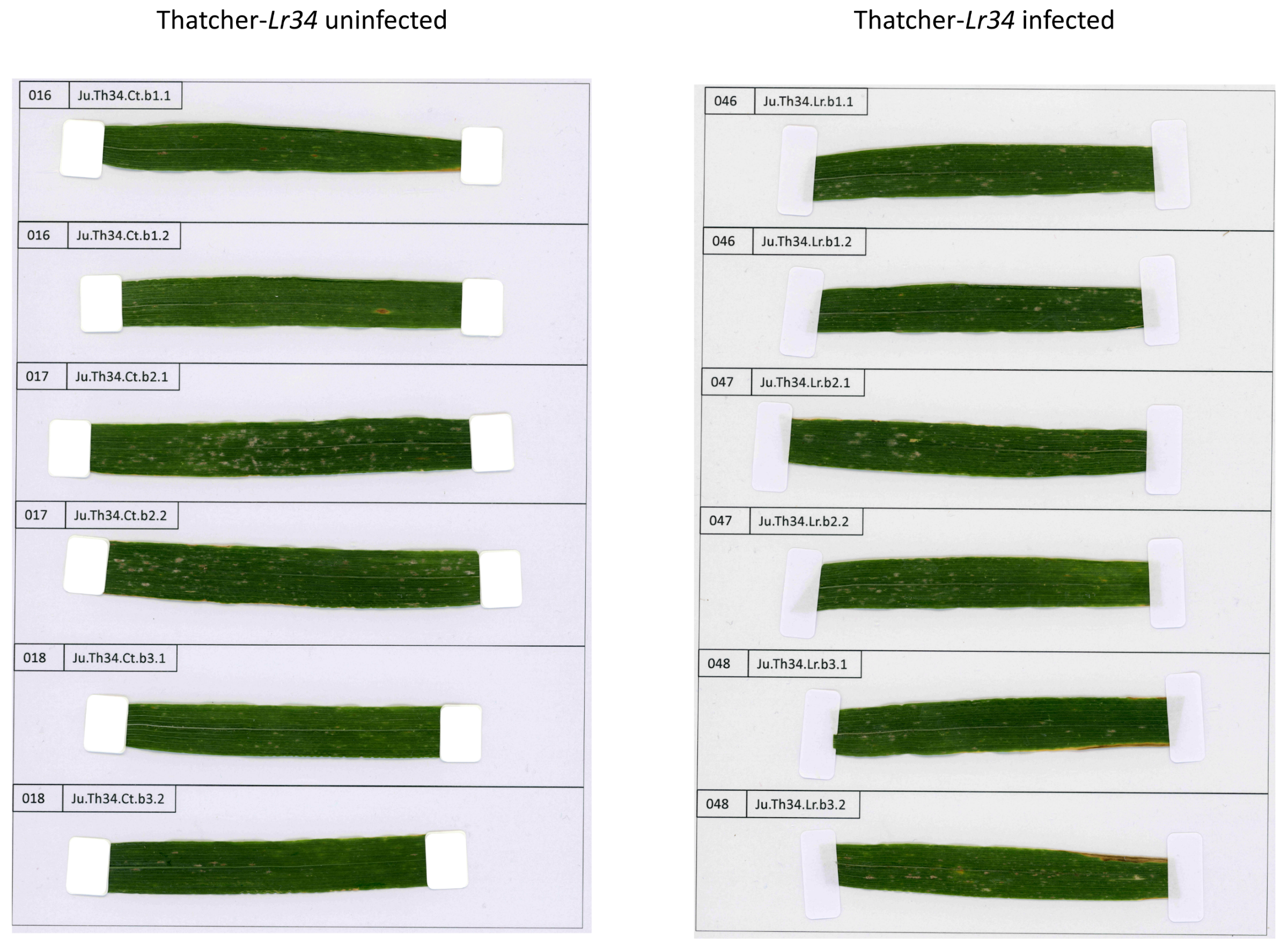


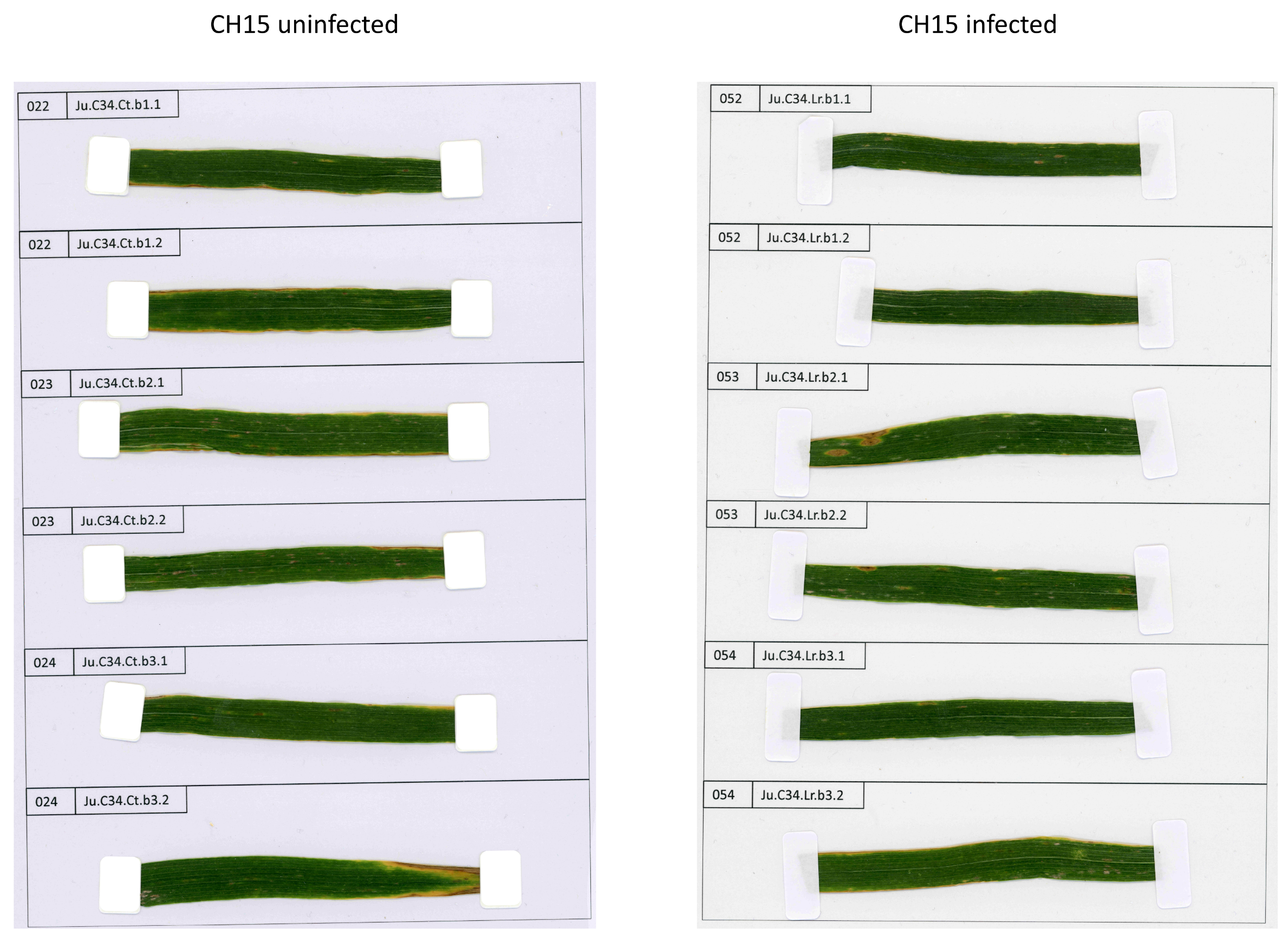


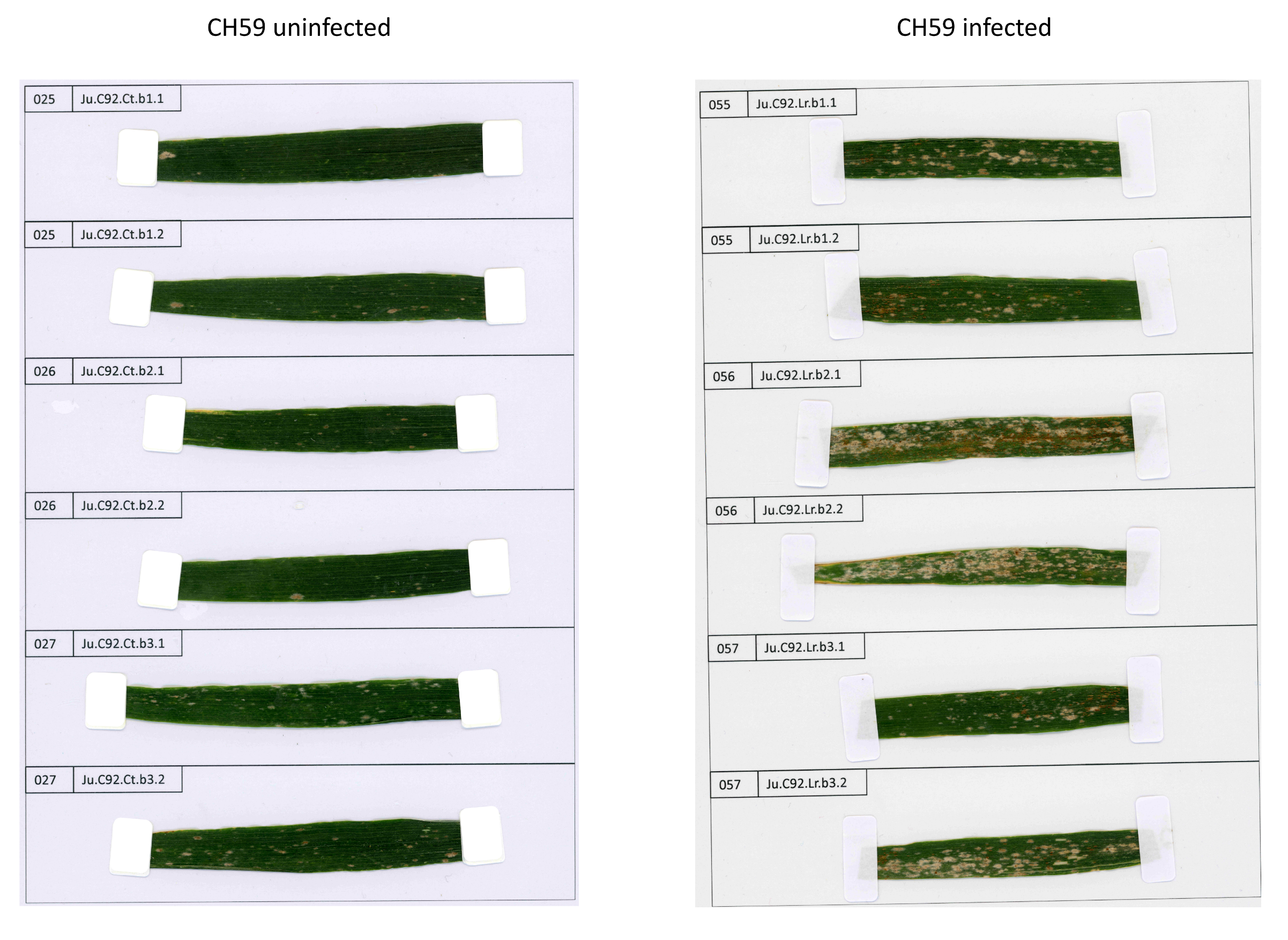


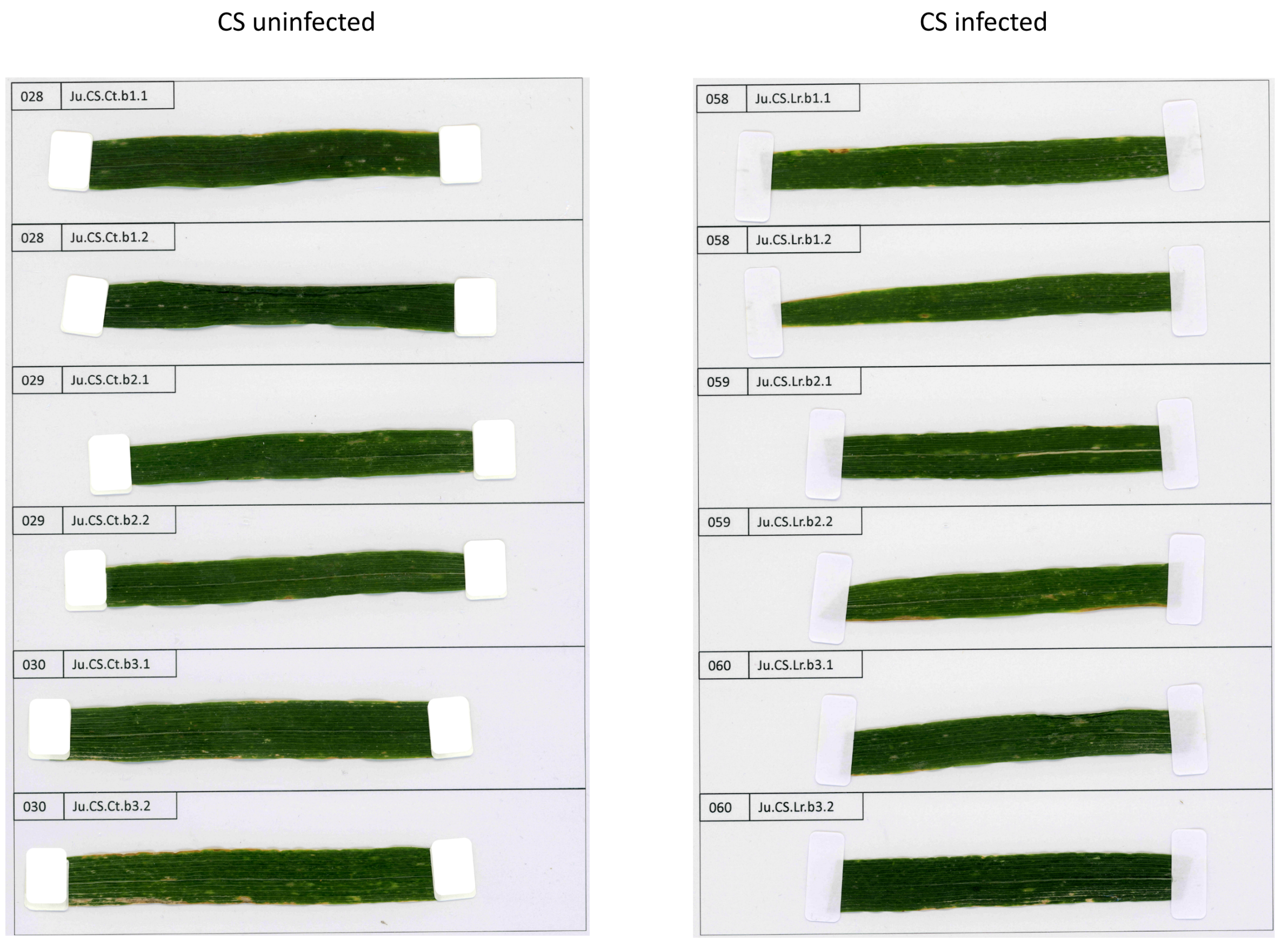


**Supplementary fig. S4. Field infection experiment.** Five different wheat lines were infected with a mixture of leaf rust fungi. Flag leaves were collected at late infection stage from test (infected) and control (uninfected) adult wheat plants. Note that symptoms of wheat powdery mildew are also visible. Wheat cultivars: Thatcher (Th), Thatcher-*Lr34* (Th34)*,* Chinese Spring (CS), AUS 27378 (CH15), AUS 27438 (CH59).


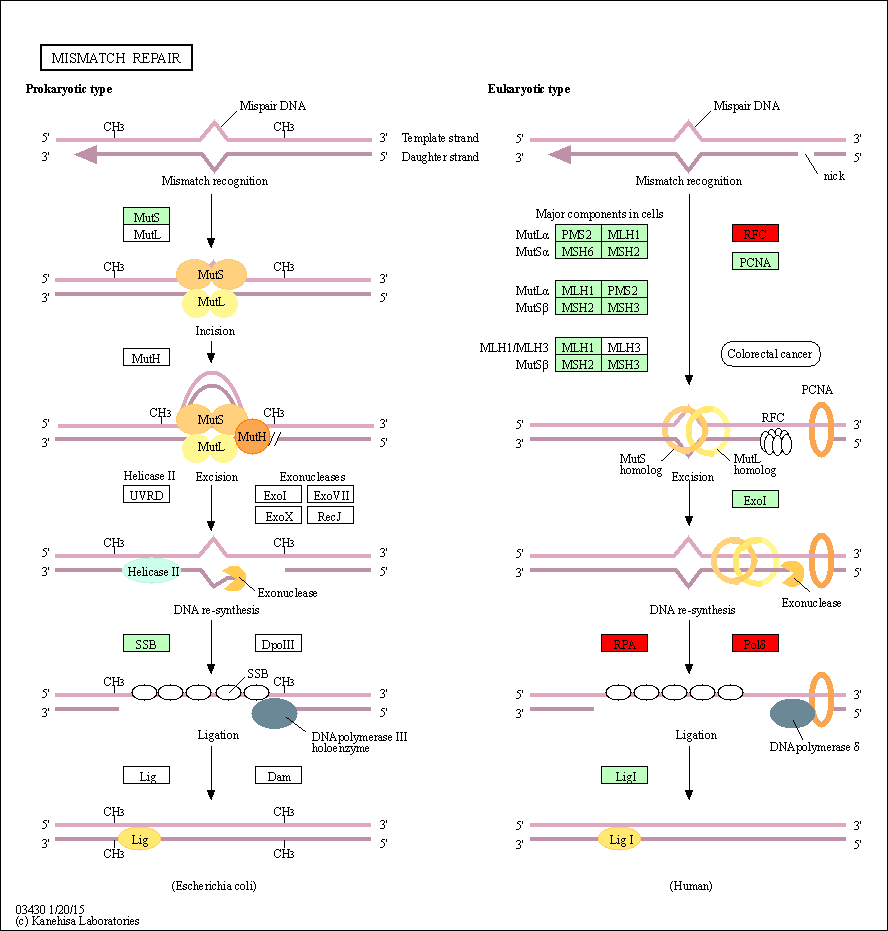


**Supplementary fig. S5.** Mismatch repair KEGG pathway (osa03430, enrichment corrected *p*-value 4.86E-03). KOBAS was used to illustrate the enrichment of genes that are down-regulated in Thatcher upon leaf rust infection. Red boxes: genes that were down-regulated; light green boxes: *O. sativa* genes that have been previously identified; white boxes: genes that belong to the KEGG pathway but have no currently identified *O. sativa* ortholog.


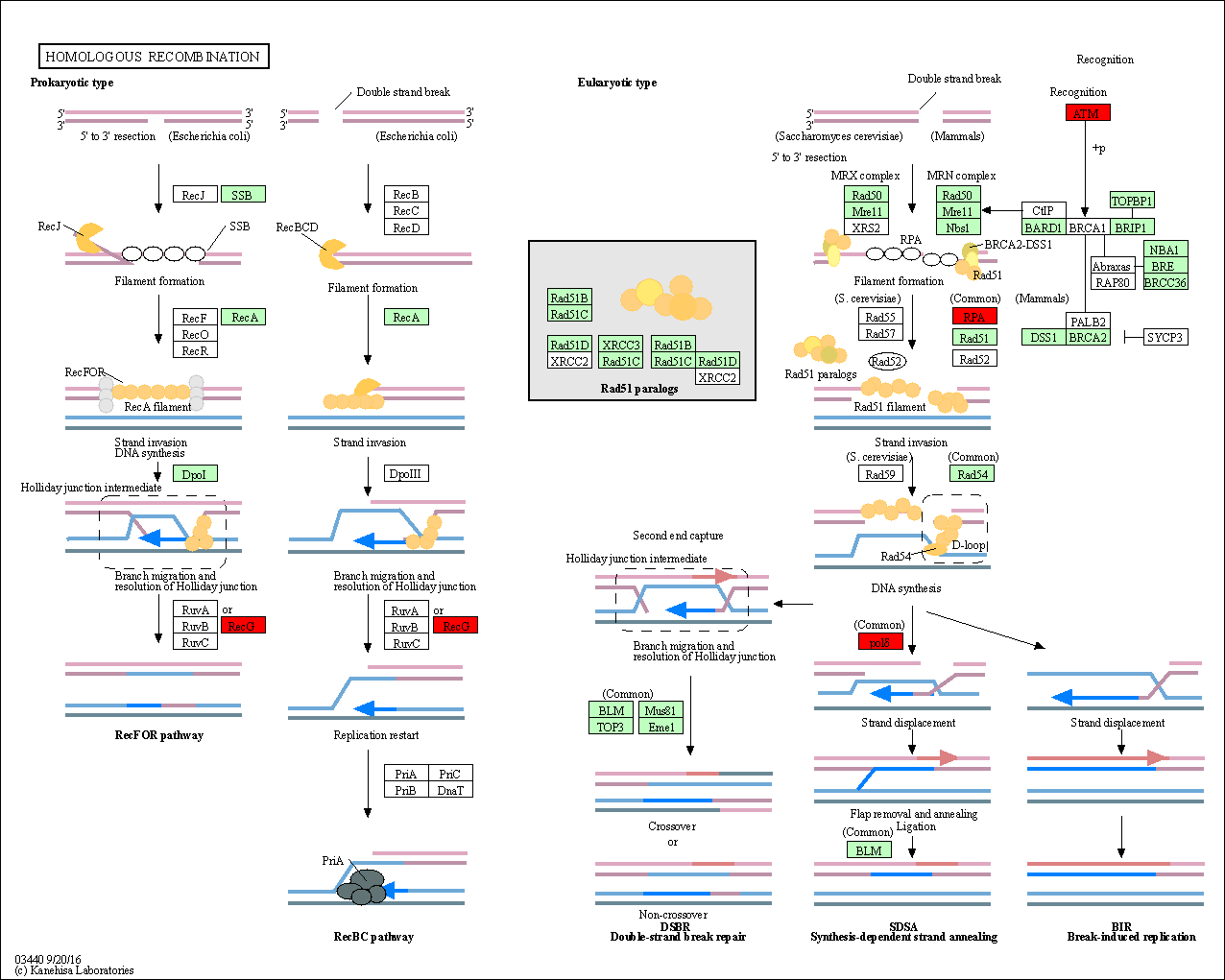


**Supplementary fig. S6.** Homologous recombination KEGG pathway (osa03440, enrichment corrected *p*-value 2.77E-02). KOBAS was used to illustrate the enrichment of genes that are down-regulated in Thatcher upon leaf rust infection. Red boxes: genes that were down-regulated; light green boxes: *O. sativa* genes that have been previously identified; white boxes: genes that belong to the KEGG pathway but have no currently identified *O. sativa* ortholog


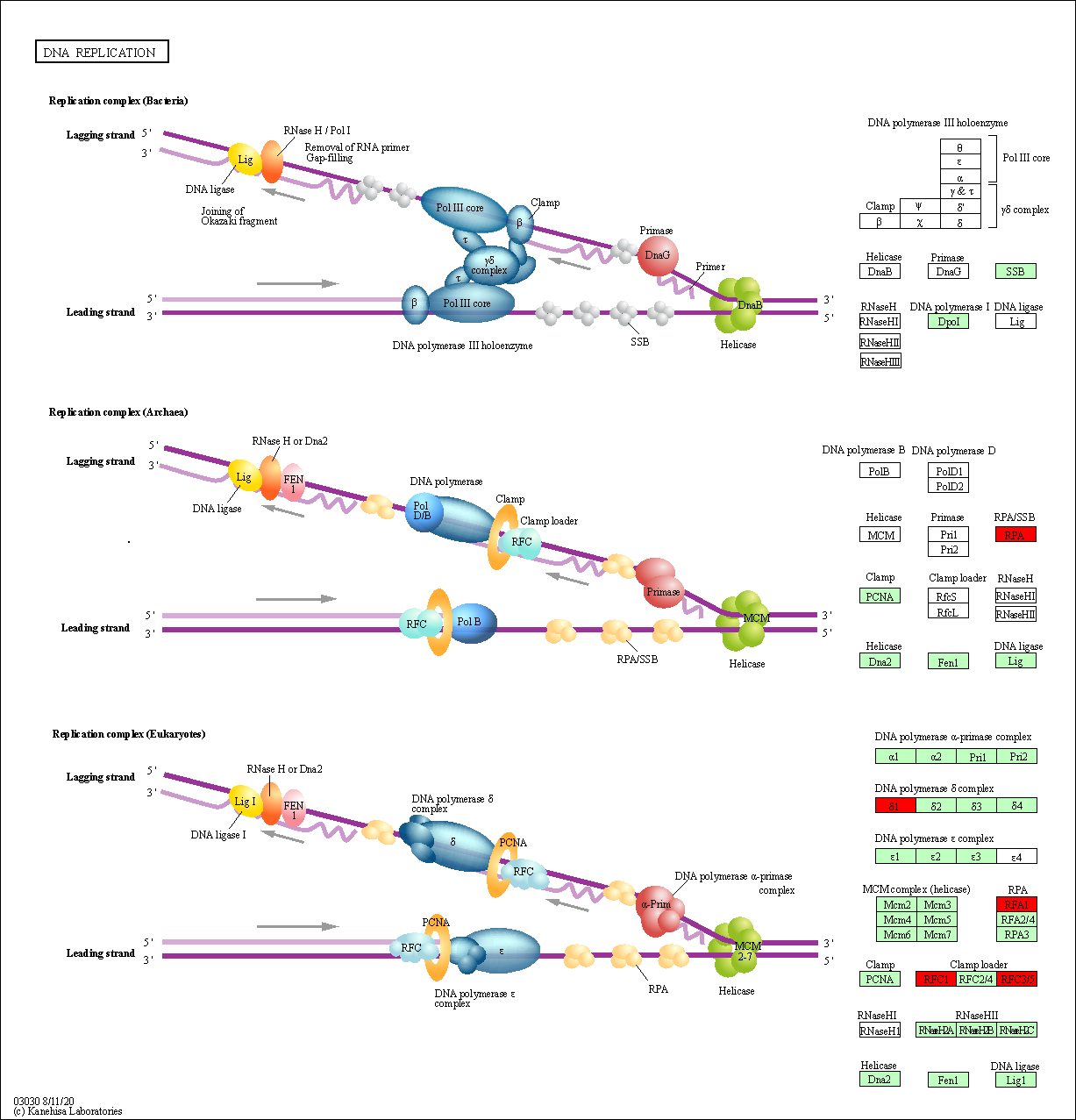


**Supplementary fig. S7.** DNA replication KEGG pathway (osa03030, enrichment corrected *p*-value 1.26E-02). KOBAS was used to illustrate the enrichment of genes that are down-regulated in Thatcher upon leaf rust infection. Red boxes: genes that were down-regulated; light green boxes: *O. sativa* genes that have been previously identified; white boxes: genes that belong to the KEGG pathway but have no currently identified *O. sativa* ortholog.


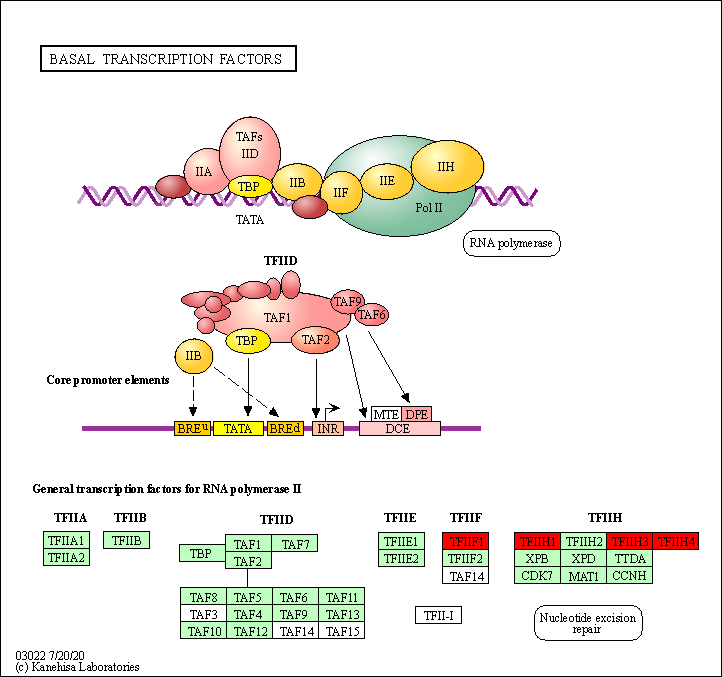


**Supplementary fig. S8.** Basal transcription factors KEGG pathway (osa03022, enrichment corrected *p*-value 4.76E-02). KOBAS was used to illustrate the enrichment of genes that are down-regulated in Thatcher upon leaf rust infection. Red boxes: genes that were down-regulated; light green boxes: *O. sativa* genes that have been previously identified; white boxes: genes that belong to the KEGG pathway but have no currently identified *O. sativa* ortholog.


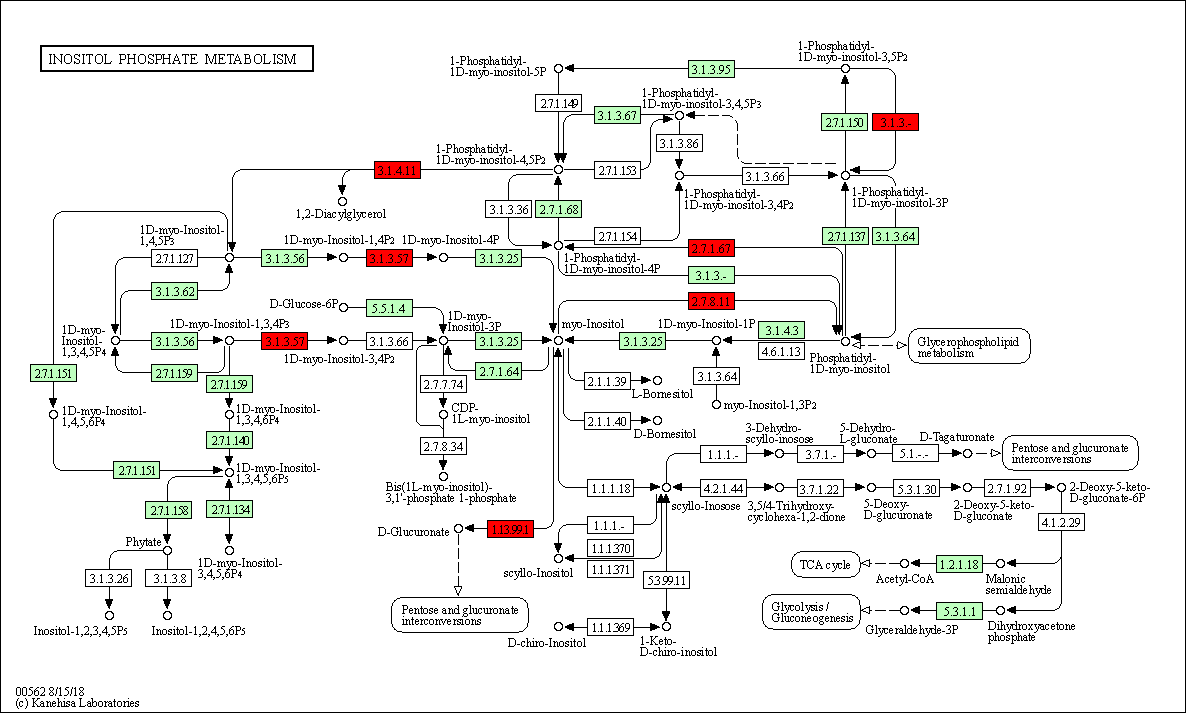


**Supplementary fig. S9.** Inositol phosphate metabolism KEGG pathway (osa00562, enrichment corrected *p*-value 3.69E-02). KOBAS was used to illustrate the enrichment of genes that are down-regulated in Thatcher upon leaf rust infection. Note that EC 3.1.4.11 indicates the enzyme phosphatidylinositol phospholipase C (PI-PLC). Red boxes: genes that were down-regulated; light green boxes: *O. sativa* genes that have been previously identified; white boxes: genes that belong to the KEGG pathway but have no currently identified *O. sativa* ortholog.


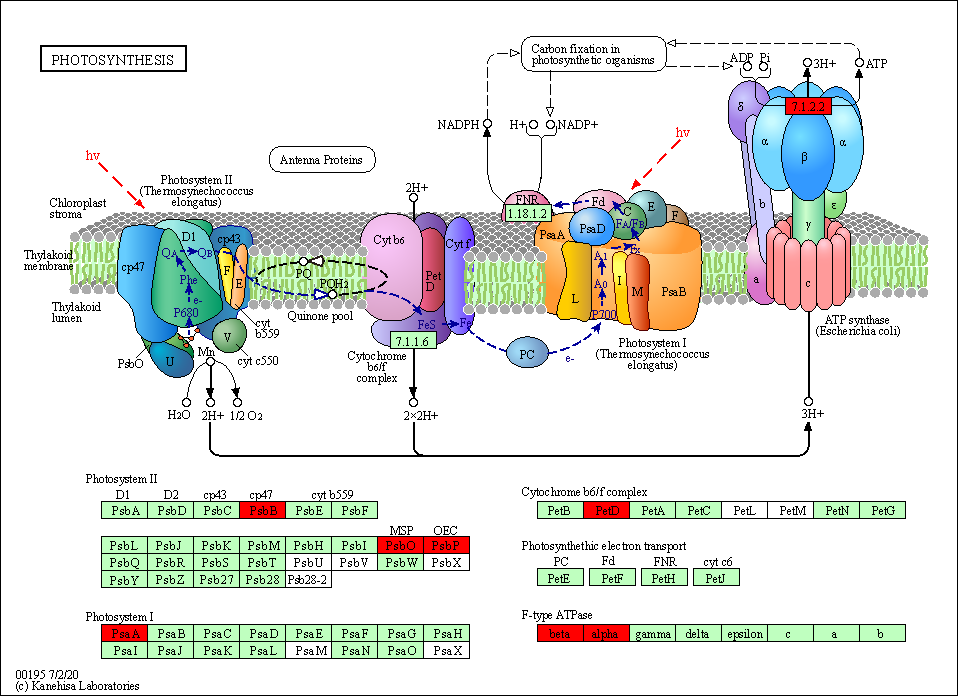


**Supplementary fig. S10.** Photosynthesis KEGG pathway (osa00195, enrichment corrected *p*-value 2.77E-02). KOBAS was used to illustrate the enrichment of genes that are down-regulated in Thatcher upon leaf rust infection. In total, this pathway contains 7 unique down-regulated genes. Red boxes: genes that were down-regulated; light green boxes: *O. sativa* genes that have been previously identified; white boxes: genes that belong to the KEGG pathway but have no currently identified *O. sativa* ortholog.
